# Proteome and Phosphoproteome Changes Associated with Prognosis in Acute Myeloid Leukemia

**DOI:** 10.3390/cancers12030709

**Published:** 2020-03-17

**Authors:** Elise Aasebø, Frode S. Berven, Sushma Bartaula-Brevik, Tomasz Stokowy, Randi Hovland, Marc Vaudel, Stein Ove Døskeland, Emmet McCormack, Tanveer S. Batth, Jesper V. Olsen, Øystein Bruserud, Frode Selheim, Maria Hernandez-Valladares

**Affiliations:** 1 Department of Clinical Science, University of Bergen, 5021 Bergen, Norway; Elise.Aasebo@uib.no (E.A.); Sushma.Bartaula@uib.no (S.B.-B.); Tomasz.Stokowy@uib.no (T.S.); Marc.Vaudel@uib.no (M.V.); Oystein.Bruserud@uib.no (Ø.B.); 2 The Proteomics Facility of the University of Bergen (PROBE), University of Bergen, 5009 Bergen, Norway; Frode.Berven@uib.no (F.S.B.); Frode.Selheim@uib.no (F.S.); 3 The Department of Biomedicine, University of Bergen, 5009 Bergen, Norway; stein.doskeland@uib.no; 4 Department for Medical Genetics, Haukeland University Hospital, 5021 Bergen, Norway; Randi.Hovland@uib.no; 5 Department of Biological Sciences, University of Bergen, 5006 Bergen, Norway; 6 Centre for Cancer Biomarkers, Department of Clinical Science, University of Bergen, 5021 Bergen, Norway; Emmet.Mc.Cormack@uib.no; 7 Novo Nordisk Foundation Center for Protein Research, University of Copenhagen, 2200 Copenhagen, Denmark; t.batth@cpr.ku.dk (T.S.B.); jesper.olsen@cpr.ku.dk (J.V.O.)

**Keywords:** acute myeloid leukemia, proteome, phosphoproteome, kinase, V-ATPase, markers, patient relapse, mass spectrometry

## Abstract

Acute myeloid leukemia (AML) is a hematological cancer that mainly affects the elderly. Although complete remission (CR) is achieved for the majority of the patients after induction and consolidation therapies, nearly two-thirds relapse within a short interval. Understanding biological factors that determine relapse has become of major clinical interest in AML. We utilized liquid chromatography tandem mass spectrometry (LC-MS/MS) to identify the protein changes and protein phosphorylation events associated with AML relapse in primary cells from 41 AML patients at time of diagnosis. Patients were defined as relapse-free if they had not relapsed within a five-year clinical follow-up after AML diagnosis. Relapse was associated with increased expression of RNA processing proteins and decreased expression of V-ATPase proteins. We also observed an increase in phosphorylation events catalyzed by cyclin-dependent kinases (CDKs) and casein kinase 2 (CSK2). The biological relevance of the proteome findings was supported by cell proliferation assays using inhibitors of V-ATPase (bafilomycin), CSK2 (CX-4945), CDK4/6 (abemaciclib) and CDK2/7/9 (SNS-032). While bafilomycin preferentially inhibited the cells from relapse patients, the kinase inhibitors were less efficient in these cells. This suggests that therapy against the upregulated kinases could also target the factors inducing their upregulation rather than their activity. This study, therefore, presents markers that could help predict AML relapse and direct therapeutic strategies.

## 1. Introduction

Acute myeloid leukemia (AML) is an aggressive and heterogeneous malignancy, the two main subsets being acute promyelocytic leukemia (APL) and the heterogenous group non-APL AML [1,2]. The APL variant is described by a characteristic clinical picture, specific cytogenetic abnormalities, different treatment than the other non-APL variants and a favorable prognosis [3]. In this article, we will use the term AML to refer to the non-APL variants of the disease, and these variants are also highly heterogeneous regarding genetic abnormalities and prognosis (i.e., relapse-free survival), with a median age at the time of first diagnosis of 65–70 years [4]. Despite the heterogeneity of the non-APL variants, these patients are treated according to the same guidelines, both with regard to intensive and potentially curative treatment (possibly including stem cell transplantation) for the young and fit subset of patients and less intensive leukemia-stabilizing treatment for elderly (i.e., above 70–74 years of age) or unfit patients [4,5,6]. Most patients receiving intensive anti-AML treatment achieve initial disease control (i.e., complete hematological remission), but a major cause of death is due to primary resistance and chemoresistant leukemia relapse either during or following the chemotherapy [4,5,7]. The overall long-term AML free survival, even for young and fit AML patients, is therefore only approximately 50% [8]. Thus, there is a need for a better prognostic classification and therapeutic strategies both for the younger patients receiving intensive therapy and for the large majority of elderly or unfit patients receiving leukemia-stabilizing therapy. 

Even though previous studies have detected associations between prognosis and the phosphorylation status of selected intracellular mediators [9,10], only cytogenetic abnormalities and submicroscopic mutations of certain genes (e.g., in *CEBPA*, *FLT3, NPM1* and *TP53*) are implemented in the routine prognostic evaluation and risk adapted treatment of AML patients procedures [8,11,12,13,14,15]. However, the underlying of AML relapse determinants remains elusive, as most of the late relapse cases are associated with a normal karyotype or with the absence of typical cytogenetic abnormalities observed in therapy-related AML [16,17]. Mutations in *NPM1* and in signaling genes such as *NRAS*, *KIT* and *PTPN11* are frequently observed at time of diagnosis but are less found at relapse [18,19]. However, *FLT3*-ITDs and mutations in *WT1*, *KDM6A* and *RUNX1* are often found at relapse [8,20]. Although epigenetic regulation might be necessary for the development of relapse, regulators of DNA methylation and of chromatin remodeling as well as histone modifiers show different evolutionary patterns from diagnosis to relapse stages [21]. A separate longitudinal genomic characterization has shown that in 80% of the patients, the founder leukemic clone survived chemotherapy and provide a basis for late relapse [22]. 

Liquid chromatography tandem mass spectrometry (LC-MS/MS)-based proteomics or phosphoproteomics have been utilized for the subclassification of patients with non-APL variants of AML [23,24,25] and for the study of proteins released by apoptosis-resistant and sensitive primary AML cells [26]. Remarkable advances in the mass spectrometry technology over the past decades have provided equipment with optimized resolution, allowing high coverage characterizations of post-translation modifications (PTMs). Thus, predictive phosphorylation markers for the treatment of AML with FLT3 inhibitor quizartinib, the description of the phosphotyrosine-proteome, tyrosine-kinome and tyrosine-phosphatome in AML, and the identification of sensitivity determinants of AML cells to kinase inhibitors have been recently described [27,28,29].

We previously showed that a super-SILAC (Stable Isotope Labeling with Amino Acids in Cell Culture) mix based on five AML cell lines provided a solid reference for quantitative proteomics studies of AML patient cells [30]. Together with optimized sample preparation and phosphopeptide enrichment protocols, our proteomics workflows proved to be useful for the study of prognosis biomarkers and treatment response in AML [31,32,33].

In the present population based-study, in order to identify relapse promoters at diagnosis, we compared the proteome and phosphoproteome profiles of pretreatment AML cells collected at the time of diagnosis for patients who later became long-term leukemia-free survivors (at least 5 years AML-free survival), or had a primary resistant disease, or suffered from a chemoresistant relapse after completing the planned intensive therapy. Based on the proteomics and phosphoproteomics analysis of these two groups, we found common denominators in pretreatment samples such as RNA processing and V-ATPase proteins, that should be further investigated as potential prognostic biomarkers or possible therapeutic targets.

## 2. Results

### 2.1. AML Patients Included in the Study

**Figure 1:**
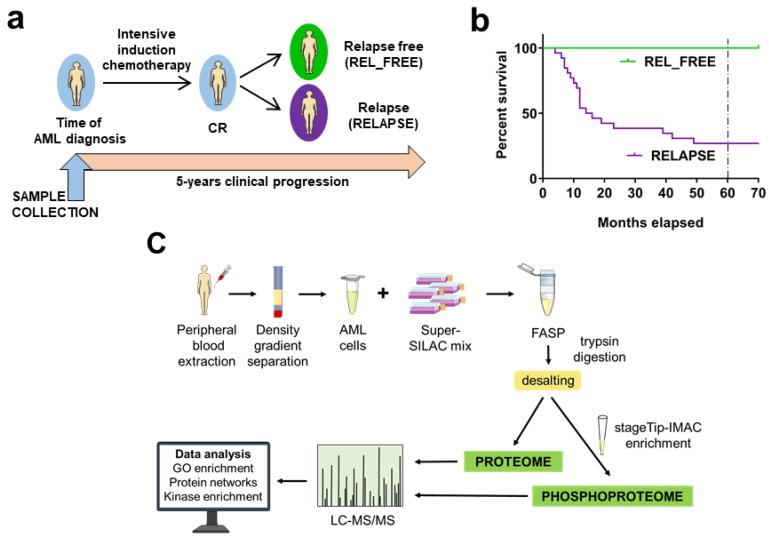
Overview of the RELAPSE and REL_FREE AML patient cohort and the workflows for the proteome and phosphoproteome analysis of acute myeloid leukemia (AML) patient cells. (**a**) The study included AML cell samples from 26 RELAPSE and 15 REL_FREE patients collected at the time of first diagnosis. All patients received intensive induction chemotherapy, consolidation therapy and achieved complete remission (CR), as described in Materials and Methods. Patients were classified after an observation time of at least five years from the initial therapy. (**b**) Survival plot for the patients included in each group. Dashed line indicates the five-years observation time. (**c**) AML sample preparation steps for proteome and phosphoproteome analysis include blood extraction, blast isolation, cell lysis, addition of super-SILAC (Stable Isotope Labeling with Amino Acids in Cell Culture) mix, filter-aided sample preparation (FASP)-based protein digestion and additional immobilized metal affinity chromatography (IMAC) enrichment of phosphopeptides. After liquid chromatography tandem mass spectrometry (LC-MS/MS) acquisition, our proteomic workflow ends with bio-computation and validation analyses.

To study the proteome and phosphoproteome changes between chemoresistant/relapse (RELAPSE) and relapse-free (REL_FREE) patients at the time of first diagnosis, we selected 41 patient samples, as illustrated in Figure 1a,b. The main characteristics of our patient cohort are given in Table 1 and Table S1. As expected, approximately half of the 41 patients showed a normal karyotype (22 out of 36 investigated patients); the most common mutations were on *NPM1* and *FLT3*-ITD (Table S1). Furthermore, when analyzing the whole patient cohort, *NPM1* mutations showed significant correlations both with morphological signs of AML cell differentiation (especially FAB M4/M5; Fischer’s exact test, *p* = 0.023) and DNA methylation gene mutations (*p* = 0.003). All these observations are consistent with observations previously described for AML in general [4,12,13,14]. Finally, the percentage of bone marrow AML blasts did not differ between the two subsets (*p* = 0.247).

Patients with and without relapse showed expected differences in the frequencies of favorable *NPM1* mutations (increased in REL_FREE patients) and adverse *FLT3*-ITD (increased in RELAPSE patients) [8]. Furthermore, REL_FREE patients also showed a high frequency of monocytic differentiation by their leukemic cells (i.e., FAB M4/M5), that was significantly different from the RELAPSE patients (Fischer’s exact test, *p* = 0.003). This is mainly due to the expected and significant association between *NPM1* mutations and morphological signs of differentiation (i.e., low frequency of FAB M0/M1 and high frequency especially of M4/M5; Fischer’s exact test, *p* = 0.023) but one inv(16) patient with expected M4 morphology also contributed. Previous studies have also described an association between *NPM1* mutations and morphological signs of differentiation and expression of the CD33 differentiation marker, as well as an inverse correlation with expression of the CD34 stem cell marker [34,35]. Thus, all observations described above are expected and can be explained by previously described characteristics of AML patient subsets in large AML studies. 

### 2.2. The Protein Abundances of rRNA Processing Proteins and V-ATPase Subunits Differ Between RELAPSE and REL_FREE Patients

We compared the proteome profiles of AML cells derived from 26 RELAPSE and 15 REL_FREE patients at the time of diagnosis, obtained according to our filter-aided sample preparation (FASP)-based workflow (Figure 1c). We quantified 6781 proteins, of which 5309 had a quantitative value in at least five patients in each group. The t-test based statistical analysis resulted in 351 differentially expressed proteins; 210 proteins were upregulated and 141 were downregulated in the RELAPSE relative to REL_FREE group (Supplementary file 1). The proteome of patient-derived cell lysates was also processed and quantified using label-free quantification (LFQ), for validating the super-SILAC based proteome quantitation (Supplementary data, Figure S1, Figure S2, Figure S3 and Supplementary file 1). The RELAPSE vs. REL_FREE fold changes (FCs) of the 4041 proteins quantified in both experiments had good correlation (Pearson *R* = 0.72; Figure S2). 

**Figure 2:**
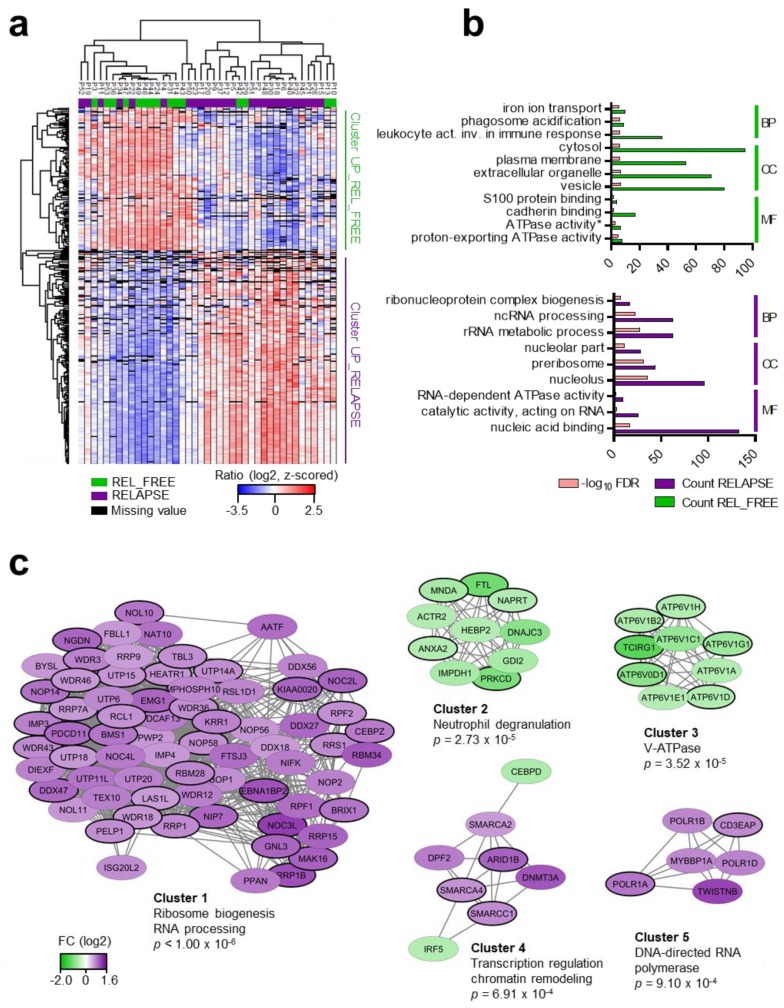
The global AML cell proteome shows increased abundance of rRNA processing proteins and decreased abundance of V-ATPase subunits for patients who relapse. (**a**) Hierarchical clustering of the patients (P1-P53), based on protein expression (SILAC log_2_ ratio) of the 351 proteins with significantly different regulation in AML cells from REL_FREE (green) and RELAPSE (purple) patients. Two vertical main clusters were observed, one dominated by proteins with higher abundance in mostly REL_FREE patients (Cluster UP_REL_FREE) and the other by proteins with higher abundance in RELAPSE patients (Cluster UP_RELAPSE). (**b**) Gene ontology (GO) analyses of the two protein clusters identified in Figure 2a were performed, to reveal enriched biological processes (BP; see the right side of the figure), cellular compartments (CC) and molecular functions (MF) in the two protein clusters. The upper part of the figure corresponds to the upper REL_FREE cluster identified in Figure 2a, whereas the lower part of the figure corresponds to the lower RELAPSE cluster. The various enriched GO terms are listed in the left part of the figure. The number of proteins associated to a specific GO term (count) and the corresponding –log_10_ false discovery rate (FDR) of these top significant GO terms are shown on the x-axis. *The complete name of the GO term referred to as ATPase activity in the figure is “ATPase activity, coupled to transmembrane movement of ions, rotational mechanism”. (**c**) Protein–protein interactions (PPI) networks of the 351 proteins from the STRING database, visualized and analyzed with Cytoscape and ClusterONE, respectively. The five clusters with highest significance of cohesiveness are shown with *p* values of a one-sided Mann–Whitney U test. The protein nodes are colored according to their RELAPSE/REL_FREE log_2_ fold change (FC), i.e., purple indicates increased abundance in the RELAPSE group and green increased abundance in the REL_FREE group. Protein nodes with black border represent significantly regulated proteins found in both the SILAC and label-free datasets.

Hierarchical clustering of the 351 differential proteins grouped the patients into two main clusters (Figure 2a), which corresponded to the RELAPSE and REL_FREE samples, although a distinct separation was not obtained. Gene ontology (GO) enrichment analysis showed that ATPase activity (coupled to transmembrane movement of ions, rotational mechanism), proton-exporting ATPase activity, phagosome acidification and iron ion transport (Figure 2b) were more abundant processes in the REL_FREE patients. These terms include proteins of the vacuolar (V)-H^+^-ATPase, an ATP-dependent proton pump in cellular membranes, such as lysosomes and endosomes [36]. The proteins enriched in this group were primarily located in the cytosol or extracellular organelles, such as vesicles. In contrast, proteins involved in ribosomal processes, such as rRNA metabolic process, ncRNA processing and ribonucleoprotein complex biogenesis were clearly higher in the RELAPSE patients, where 46% of the increased proteins were annotated to the nucleolus in the cellular compartment analysis (Figure 2b). 

Moreover, a protein-protein interaction (PPI) network of the differentially expressed proteins (Figure 2c) confirmed the increased abundance of proteins involved in rRNA processing and ribosome biogenesis in relapsed patients (cluster 1; 69 proteins). RRP1B (*p* < 0.0002, FC 1.09) and AATF (*p* < 0.0002, FC 0.91) were the most significant proteins in this cluster. AATF (also known as CHE1) participates in several cellular pathways, such as 40S ribosomal subunit synthesis in complex with NGDN (*p* = 0.0014, FC 0.95) and NOL10 (*p* = 0.0007, FC 0.85) [37], and high AATF expression has been linked to variants of leukemia [38,39]. Other proteins in this cluster were CEBPZ, a transcription factor in the CEBP family, several WD repeat-containing proteins (e.g., WDR3, WDR18, WDR36), probable ATP-dependent RNA helicases (e.g., DDX18, DDX27, DDX56) and members of the C/D box small nucleolar ribonucleoprotein (snoRNP) complex (NOP56 and NOP58), which guides 2’O methylation of the rRNA. Proteins involved in neutrophil degranulation dominated cluster 2 and nine subunits belonging to the V-ATPase complex were identified in cluster 3 (Figure 2c), all with higher abundance in the REL_FREE patients. Regulated V-ATPase subunits (cluster 3) and proteins participating in rRNA processing and ribosomal biogenesis (cluster 1) are detailed in Supplementary file 2. Cluster 4 and 5 contained proteins involved in chromatin remodeling and RNA polymerase I subunits, respectively, which were upregulated in the RELAPSE patients. 

The gene set enrichment analysis (GSEA) against the Hallmark gene set collection, conducted to identify classes of genes overrepresented in the complete dataset, resulted in HALLMARK_MYC_TARGETS_V2 (systematic name: M5928), as the only significant gene set (false discovery rate, FDR, *q*-value = 0.046) (Figure S4). This gene set was upregulated in the RELAPSE group and consisted of 58 genes [40]. A total of 15 out of the 30 proteins who contributed most to the gene set enrichment results (i.e. the leading edge subset) were found in cluster 1 in Figure 2c.

Using the data on time from diagnosis to relapse (Table S1), we further classified the RELAPSE patients into two groups: 14 EARLY RELAPSE and 12 LATE RELAPSE patients with first relapse occurring before and at/after 12 months from diagnosis, respectively, in alignment with reported remission duration [41,42]. We found 236 and 295 differentially expressed proteins in the EARLY RELAPSE vs. REL_FREE and in the LATE RELAPSE vs. REL_FREE comparisons, respectively. One hundred and twelve regulated proteins from each set overlapped and some of these belonged to the ribosome biogenesis RNA processing, the DNA-directed RNA polymerase and V-ATPase clusters that we identified when we analyzed all the RELAPSE patients together (Figure S5, Figure 2c). Thus, although the EARLY and LATE RELAPSE proteomes might show some features related to the different relapse timings, regulation of relevant protein networks were observed in all relapsed patients.

### 2.3. Differential CDK, CSK2 and PRKCA/D Kinase Activities between RELAPSE and REL_FREE Patients

We constructed a dataset comprising 12,309 identified and quantified class I protein phosphorylation sites from 3003 proteins of 26 RELAPSE and 15 REL_FREE patients. Serine phosphorylation made up the majority of the identified phosphosites (89.7%) while phosphorylation on threonine and tyrosine (10.0% and 0.3%, respectively) comprised the rest. We identified 274 differentially regulated phosphorylated sites based on a statistical analysis of 5634 phosphosites, which were quantified in at least five patients in each group (Supplementary file 3). 

**Figure 3:**
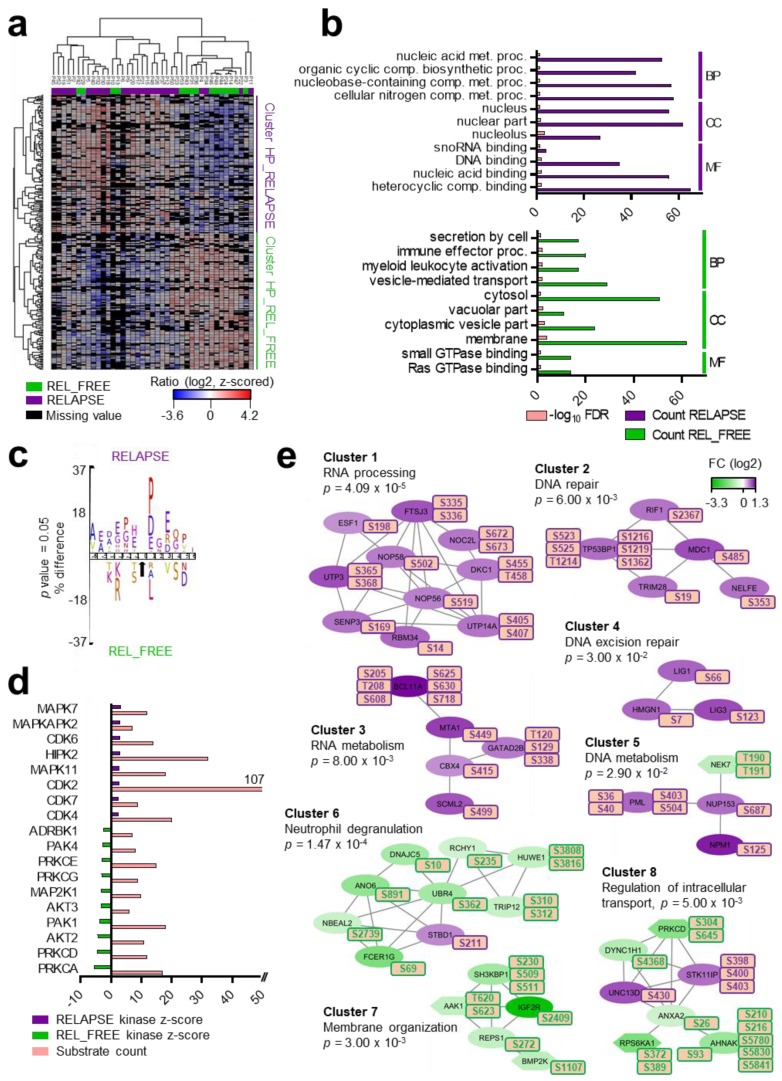
The AML RELAPSE phosphoproteome is enriched in cyclin-dependent kinase (CDK) substrates and RNA processing casein kinase 2 (CSK2) targets. (**a**) Hierarchical clustering of 274 differentially regulated phosphorylation sites revealed two clusters: HP (High Phosphorylation)_RELAPSE (in purple) and HP_REL_FREE (in green). The SILAC log_2_ ratio scale and color code is also shown. (**b**) GO analyses of the two phosphoprotein clusters identified in Figure 3a were performed to reveal enriched BP, indicated to the right in the figure, CC and MF. The GO terms are listed in the left part of the figure; the upper part of the figure corresponds to the upper RELAPSE cluster identified in Figure 3a whereas the lower part of the figure corresponds to the lower REL_FREE cluster. The x-axis indicates the number of phosphoproteins/FDR. (**c**) Sequence motif analysis of the ± six amino acids, flanking the differentially regulated phosphorylation sites for either cluster. (**d**) Kinase-substrate enrichment analysis (KSEA) of differentially regulated and unregulated phosphorylation sites. The kinase z-score (X axis) is the normalized score for each kinase (Y axis), weighted by the number of identified substrates. (**e**) Networks of PPI based on STRING database and visualized in Cytoscape after ClusterONE analysis. Significance of networks of high cohesiveness is shown with the *p* value of a one-sided Mann–Whitney U test. The differentially regulated phosphorylation site(s) is shown next to each protein. FC of phosphorylation are color-coded; purple-colored proteins showed a higher phosphorylation in the RELAPSE group and green-colored proteins showed a higher phosphorylation in the REL_FREE group. Kinases are specifically distinguished using hexagon shapes.

Hierarchical clustering using these 274 phosphosites divided the phosphoproteome of RELAPSE and REL_FREE patients (Figure 3a). Two clusters, one containing 138 phosphosites and another with 136 phosphosites, were upregulated and downregulated in the RELAPSE, relative to REL_FREE patients, respectively (Supplementary file 3). 

Cellular component GO analysis revealed an enrichment of upregulated nuclear phosphoproteins for RELAPSE patients, whereas cytoplasm, cytosol, membranes and vacuolar structures were enriched in the REL_FREE group (Figure 3b). Immune effector process, myeloid leukocyte activation and GTPase binding were the biological processes and molecular functional GO terms enriched in this group. 

To identify protein kinases differentially activated in the two groups, we performed a phosphorylation site motif analysis (IceLogo) [43]. We found a strong bias towards acidic amino acids such as glutamic acid (E), in close proximity to the differentially phosphorylated sites in the RELAPSE group when compared to the unregulated phosphosites (Figure S6a), suggesting a higher casein kinase 2 (CSK2) activity in this group. Using differentially phosphorylated sites in the REL_FREE group, the analysis identified a basophilic KXpS/pT motif, suggesting a moderate activation of protein kinase C types, PRKCA and PRKCD (Figure S6b). Moreover, proline-directed motifs for phosphorylation by mitogen-activated protein kinases (MAPKs) and cyclin-dependent kinases (CDKs) were underrepresented in the REL_FREE group. When we directly compared the sequences surrounding the differentially regulated phosphorylation sites in the RELAPSE and REL_FREE groups, higher CSK2, MAPK and CDK activation was observed in the RELAPSE, and higher PRKCA/D in the REL_FREE group, confirming the previous motif analysis (Figure 3c, Supplementary file 4). Furthermore, the kinase-substrate enrichment analysis (KSEA) [44,45], which is based on phosphorylation FCs to estimate kinase’s activity, confirmed the higher activity of several MAPKs and CDKs in the RELAPSE group and the higher activity of PRKCA, PRKCD and AKT serine/threonine kinases in the REL_FREE group (Figure 3d). We found nine phosphosites on six different protein kinases in the data set of 274 differentially regulated phosphorylation sites. Seven of the nine phosphosites (including those on PRKCD, PAK2, MAP3K3, BMP2K and NEK7 kinases) were found to be upregulated in the REL_FREE group relative to the RELAPSE group. In particular, sites on the kinase domain activation loop of NEK7 (T190 and T191) were more abundant in the REL_FREE group. Conversely, phosphorylation of the receptor-type tyrosine-protein kinase FLT3 on S759 and S762 was higher in the RELAPSE group relative to the REL_FREE group. 

Several PPI networks of significant cohesiveness were found after a ClusterONE analysis based on STRING interactions of differentially phosphorylated proteins (Figure 3e). Even if stringent criteria in STRING was used, we should point out that false positives could occur in the networks. The most significant network (cluster 1) consisted of ten phosphoproteins with higher phosphorylation in RELAPSE patients, most of them being RNA binding proteins. A sequence logo analysis [46] of the amino acids surrounding the 15 phosphosites (corresponding to the ten proteins) suggested a higher activation of CSK2 in this cluster (Figure S7a). Other significant clusters included DNA binding proteins (clusters 2 and 4) and proteins involved in nucleic acid metabolism (clusters 3 and 5). A sequence logo analysis of the amino acids surrounding the phosphosites in clusters 2, 3 and 5 showed an enrichment of MAPK substrates (Figure S7b–d), although NPM1 S125 in cluster 5 is phosphorylated by CSK2 [47], and amino acid sequences around HMGN1, LIG1 and LIG3 phosphosites in cluster 4 suggested the involvement of other kinases, such as CSK2 and CDKs [48,49]. Finally, transcription factor BCL11A, which functions as a myeloid proto-oncogene, and DNA repair protein TP53BP1 showed higher phosphorylation on multiple sites in the RELAPSE group relative to the REL_FREE group. PPI networks that showed higher phosphorylation in the REL_FREE group were involved in neutrophil degranulation (cluster 6), membrane organization (cluster 7) and intracellular transport (cluster 8). Sequence logo analysis of the phosphosite motifs in these clusters suggested a higher activation of PRKCD/A kinases (Figure S7e–g). 

To further elucidate the molecular source of AML relapse in these patients, we utilized the proteome and phosphoproteome data in order to determine transcription factors responsible for driving relapse. Specifically, proteins involved in DNA transcription that were regulated at both the proteome or phosphoproteome level in the RELAPSE group were compared to ChIP-seq data from the K562 cell line (stored in the ENCODE database), in order to find the potential binding sites of transcription factors (Table S3). We identified six transcription factors (AFF1, CEBPZ, ETV6, IKZF1, PML and TRIM28) that showed the predicted regulation of proteins involved in DNA and RNA binding proteins, damaged DNA binding proteins, ribosome and carcinogenesis pathways based on GO molecular function terms and KEGG pathways analysis (Figure S8).

To explain the generally increased phosphosite regulation in the RELAPSE group, we compared the protein expression to the 274 differentially regulated phosphosites (corresponding to 169 phosphoproteins). We found that 107 (63%) phosphoproteins were not significantly changed at the protein level (Supplementary file 3). Among them, we noticed all the phosphoproteins of the DNA repair network (cluster 2) in Figure 3e (Figure S9). In contrast, we spotted 34 proteins significantly regulated at both protein and phosphosite level, including six phosphoproteins of the RNA processing network (cluster 1) in Figure 3e (Table S4 and Figure S10). For the majority of these phosphosites (including FLT3 and PML), the phosphorylation levels correlated closely with their protein expression levels. Taken together, while RELAPSE AML cells had higher expression of several phosphorylation-prone proteins, nearly 50% of the differentially regulated phosphorylation sites could not be explained by protein expression changes, suggesting increased kinase-specific phosphorylation.

In order to study the phosphorylation landscape of early and late relapsed patients separately, we reanalyzed the phosphoproteome dataset, classifying the RELAPSE into EARLY RELAPSE or LATE RELAPSE patients, as we did with the proteome dataset. We found 211 and 164 differentially regulated phosphorylated sites in the EARLY RELAPSE vs. REL_FREE and in the LATE RELAPSE vs. REL_FREE comparisons, respectively. Only 34 differentially regulated phosphosites were found in both subcohorts (Figure S11a). A PPI network analysis found components of some of the phosphoprotein clusters with higher phosphorylation in RELAPSE (clusters 1, 2 and 3) and with higher phosphorylation in REL_FREE group (clusters 6 and 8), identified when we analyzed all the RELAPSE patients together (Figure 3e) in the EARLY RELAPSE vs. REL_FREE dataset, whereas a new mixed cluster composed of RNA metabolism phosphoproteins, transcriptional regulators and SUMOylate target proteins was found in the LATE RELAPSE vs. REL_FREE dataset (Figure S11b,c). These results showed the differentiation of phosphorylated signatures in patients with early and late relapse at diagnosis.

### 2.4. V-ATPase, CSK2, CDK2/7/9 and CDK4/6 Inhibitors Affect the Proliferation of AML Cells

**Figure 4:**
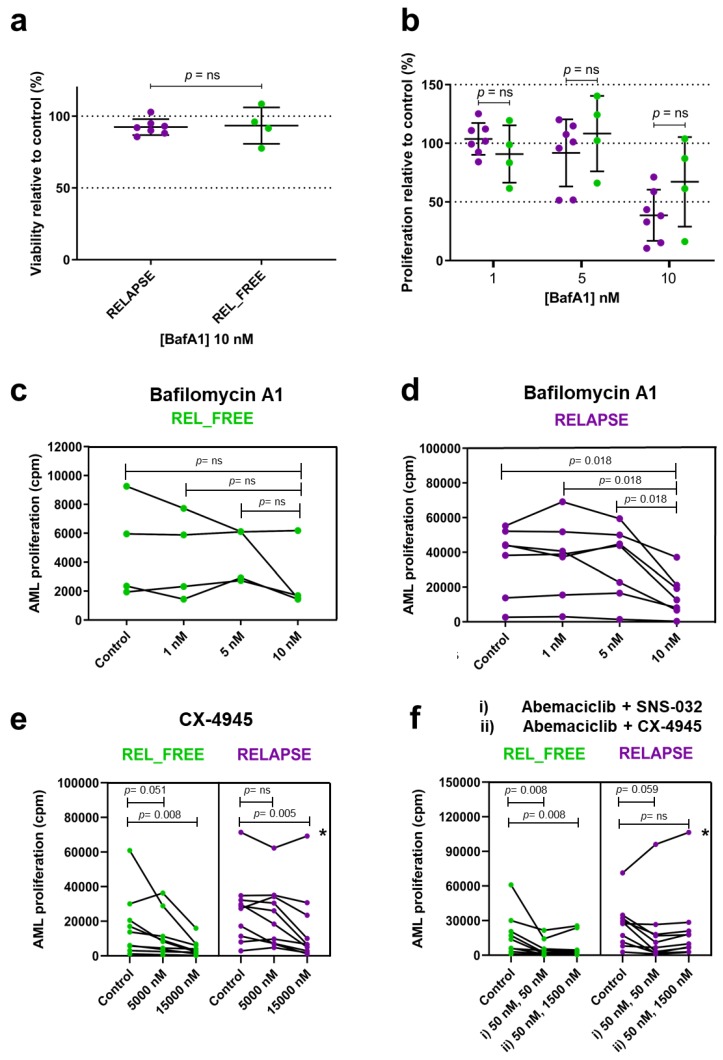
The effect of inhibitors of V-ATPases, casein kinase 2 (CSK2) or cyclin-dependent kinases (CDKs) on AML cell proliferation. AML patient cells were treated for six days with the indicated inhibitors. The thymidine incorporation-based proliferation measurements are presented as dots (purple for RELAPSE and green for REL_FREE) in patient groups or in individual patient curves. Significance of inhibitor treatment between patient groups and of inhibitor treatment vs. untreated control was found using the Mann–Whitney U test and the Wilcoxon matched-pair signed rank test, respectively. Non-significant results are indicated with “*p* = ns”. (**a**) Viability of cells relative to untreated control for RELAPSE and REL_FREE groups, when treated with 10 nM of V-ATPase inhibitor Bafilomycin A1 (BafA1). (**b**) Differences of cell proliferation relative to control for RELAPSE and REL_FREE groups when treated at 1, 5 and 10 nM BafA1. (**c**,**d**) Cells from four REL_FREE and seven RELAPSE patients treated with BafA1. The RELAPSE, but not the REL-FREE, patients had decreased proliferation (measured after 6–7 days of culture) when treated with 10 nM BafA1 inhibitor compared to control, 1 and 5 nM. Non-significant change was observed between control and 1 nM or 5 nM BafA1 inhibitor for either groups. (**e**,**f**) Cells from nine REL_FREE and ten RELAPSE patients (five and four of them were external to the patient cohort, respectively) were treated with CSK2, CDK2/7/9 and CDK4/6 kinase inhibitors in cell proliferation assays, alone or in combination. The CSK2 inhibitor CX-4945 was given at 0 (i.e., control), 5000 and 15000 nM alone. The CDK4/6 inhibitor Abemaciclib (50 nM) was given in combination with i) the CDK2/7/9 inhibitor SNS-032 (50 nM) and ii) CX-4945 (1500 nM). The same RELAPSE patient with high cell proliferation is represented with an asterisk (*).

To explore the potential of V-ATPase inhibition in AML, we tested the effect of the V-ATPase inhibitor bafilomycin A1 (BafA1) in pilot dose-response experiments and observing the growth of AML cells from four REL_FREE and seven RELAPSE patients, with high or low abundance of V-ATPase subunits, respectively (Figure S12a). We found non-significant differences in cell viability for RELAPSE and REL_FREE groups when treated at 10 nM BafA1 and in cell proliferation when treated with 1, 5 and 10 nM BafA1 (Figure 4a,b), although the inhibitor had a smaller effect on proliferation of REL_FREE cells when used at 10 nM. The proliferation was significantly higher in the untreated control RELAPSE cells compared to REL_FREE cells (Figure S12b). However, BafA1 at 10 nM decreased significantly (*p* = 0.018) the proliferation of cells for most of RELAPSE patients, whereas the proliferation of cells for three out of four REL_FREE patients was not significantly altered (Figure 4c,d). 

In order to further investigate whether the activity of the predicted RELAPSE-activated kinases was essential for cell survival and proliferation, we tested the proliferation in available AML cells from ten RELAPSE and nine REL_FREE patients in the presence of inhibitors of CSK2, CDK2/7/9, CDK4/6 and ERK1/2, alone or in combination (Table S5). As shown in Figure 4e, the CSK2 inhibitor CX-4945 at 5000 nM seemed to be slightly more potent inhibitor of REL_FREE cells compared to RELAPSE cells. Similarly, the combination of CDK4/6 inhibitor abemaciclib (50 nM) with CDK2/7/9 and CSK2 inhibitors (SNS-032 50 nM; CX-4945 1500 nM) decreased cell proliferation significantly only for the REL_FREE cells (*p* = 0.008) (Figure 4f). The RELAPSE cells showed significant antiproliferative effect only when exposed to a high concentration (15000 nM) of CX-4945 inhibitor. 

These cell proliferation studies with the kinase inhibitors showed that the RELAPSE group included an exceptional patient (marked with * in Figure 4e,f). When leaving this patient out from the statistical comparison of CX-4945 monotherapy, the same differences remained statistically significant as when all patients were included (see Figure 4e right part). However, when analyzing the effects of abemaciclib combinations without this exceptional RELAPSE patient, we obtained a *p* value of 0.008 for the inhibitor-free control vs. abemaciclib + SNS-032, whereas this *p* value was not significant 0.059 if all RELAPSE patients were included (see Figure 4f right part). Furthermore, a *p* value of 0.038 was seen if this patient was excluded from the comparison of inhibitor-free control vs. abemaciclib + CX-4945 (not significant when all RELAPSE patients were included, see Figure 4f right part). AML is a heterogeneous disease and the detection of single exceptional patients is, in our opinion, expected. However, even when leaving out this exceptional RELAPSE patient, the antiproliferative effects were generally stronger for REL_FREE than for RELAPSE patients when testing abemaciclib + SNS-032 (median proliferation in percent of the inhibitor-free control cultures being 36% with range 11–97% for REL_FREE vs. median 41% with range 10–72% for RELAPSE patients) and abemaciclib + CX-4945 (median 41% with range 19–85% for REL_FREE vs. median 63% with range 17–104% for RELAPSE patients).

Western blot analyses using lysates from three RELAPSE and three REL_FREE patient cells incubated with media (control) or CDK2/7/9 or ERK1/2 inhibitors showed both higher CDK2 expression and higher phosphorylation on T160 in the RELAPSE group, although differences between groups were only significant for the phosphorylated protein in the control samples and after treatment with SNS032 inhibitor (Figure S13b). SNS032, a compound that targets the kinase catalytic ATP-binding pocket [50], did not affect CDK2 T160 phosphorylation in any group, although this might be due to the short incubation time (15 min) used in the experiment [51,52]. The phosphorylation of ERK1/2 was low and similar in the two patient groups. SCH772984, an ATP-competitive compound of ERK1/2 [53], decreased kinase activity similarly in the RELAPSE and REL_FREE samples. These findings correlate with the phosphoproteome analyses, showing a higher relative abundance of phosphorylated CDK2 substrates, but not of ERK1/2 substrates, in the RELAPSE group.

## 3. Discussion

Chemoresistant disease (i.e. primary resistance or chemoresistant relapse) is a major cause of death in AML [8]. In this study, we compared the proteomic profiles at the time of first diagnosis for patients with later relapse and patients without leukemia relapse, after an observation time of at least five years from the initial intensive and potentially curative induction and consolidation therapy. 

Many of the statistically significant proteomic differences between patients with and without leukemia relapse were relatively small, and for this reason we did not only focus on statistically significant differences of single proteins, but also on proteomic/phosphoproteomic profiles and the similarities in biological functions for groups of differing proteins. However, we want to emphasize that the biological significance of the observed proteomic differences can only be proven by additional empirical testing. Furthermore, our present study should be regarded as a population-based study; treatment-related mortality or morbidity do not influence our results because we excluded those patients who died from or could not complete the planned intensive treatment due to severe treatment-related toxicity.

A detailed methodological discussion is therefore necessary to ensure correct and careful interpretation of our results. Our study included patients with a relative high percentage of AML blasts among circulating leukocytes. This selection was necessary to prepare highly enriched AML cell populations by a simple and standardized gradient separation alone. This strategy was chosen because more extensive cell separation procedures can alter the molecular profiles of AML cells. The possible consequences of these alterations have been discussed in detail previously [54,55]. Firstly, we previously compared the characteristics of patients selected according to these criteria (corresponding to approximately half of the consecutive patients), with the other patients without high peripheral blood blast counts during a defined time period, and we could not detect any significant differences with regard to karyotype or *FLT*3 mutations [56]. Secondly, in a previous study including 71 patients selected according to these criteria, we observed an expected frequency of various submicroscopic mutations in 54 genes frequently mutated in AML [12,13,14,34], that is also recognized in our present patient cohort (Table S1). Finally, our two patient subsets with and without relapse after completed intensive therapy are defined by very simple criteria, and the observation time used to define relapse-free patients is long. Thus, our population-based patient cohort does not differ from other larger AML patient cohorts, with regard to karyotypes and submicroscopic mutational frequencies, and the two patient subsets are defined by simple criteria based on in vivo chemosensitivity. Moreover, the association between proteomic patterns and clinical indicators such as genetic mutations and cytogenetics has been recently shown to be limited in AML suggesting proteomic-defined signatures as independent prognostic factors [57]. Therefore, further stratification analysis of the AML samples based on their FAB classification or genetic mutations might not provide additional prognostic information.

The peripheral blood blast level may have an adverse prognosic impact in AML patients receiving intensive chemotherapy, although this impact is very weak compared with karyotype and genetic abnormalities [58]. In a previous HOVON/Sakk clinical study, leukocytosis exceeding 2 × 10^9^/L was regarded as a risk factor for patients with t(8;21) [59], but this could not be confirmed in a recent separate study [60]. Furthermore, the most recent ELN guidelines do not recommend the use of peripheral blood blast counts in the prognostic evaluation of AML patients either [8]. Despite the similarities in genetic abnormalities, it can therefore be argued that we studied a patient cohort with increased chemoresistance compared with AML in general, and not only for patients with high blood blast counts. Recent studies also suggest that the leukocyte count may have a prognostic impact, but only in the small subset of patients with leukocyte counts exceeding 50 or even 100 × 10^9^/L [59,61,62]. Only six out of our 41 patients had leukocyte counts exceeding 100 × 10^9^/L and most patients had counts below 50 × 10^9^/L. Thus, we cannot exclude that the observations in our present in vivo chemotherapy study are representative only for patients with circulating AML blasts, but our selection of patients with a high percentage of circulating blasts probably represents only a minor difference from AML in general with regard to in vivo chemosensitivity. 

Our proteomic and phosphoproteomic study was based on peripheral blood and not bone marrow cells, even though AML is by definition a bone marrow disease [2], but for several reasons we regard peripheral blood cells to be relevant. First, flow cytometric studies of molecular profiles have only detected limited quantitative but not qualitative differences between peripheral blood and bone marrow cells from the same patients [63]. Recently, the reverse phase protein array (RPPA)-based proteomic profiling of blood-derived AML samples were found to be similar to that of marrow-derived samples [57]. Second, the fundamental AML associated genetic abnormalities are also detected in the peripheral blood cells. Third, peripheral blood cells also have a hierarchical organization and include cells capable of long-term in vitro proliferation (i.e., leukemic stem cells) [64] and functional comparisons of blood and bone marrow AML cells from the same patient also suggest that they show relatively small differences [65]. Finally, the definition used for most patients for the diagnosis of AML is the detection of at least 20% blasts in the bone marrow [2]. More extensive cell separation procedures would therefore be required to achieve highly enriched AML bone marrow cell populations and such separation procedures may then alter the cell characteristics [55]. 

The final methodological question is the use of cryopreserved total AML cell populations for our studies. Even though cryopreservation and thawing would be expected to influence proteomic and phosphoproteomic profiles in AML cells, our previous methodological studies with comparison of paired (i.e., both samples derived from the same patient) cryopreserved and fresh AML cell samples suggested that this influence is limited [31]. All samples in our study were cryopreserved by using the same standardized technique and without any difference in storage time between our two patient subsets. Furthermore, both our present in vivo chemotherapy study and several previous patient studies have demonstrated that the biological characteristics of the total hierarchical AML cell population reflect the risk of relapse (i.e., the chemosensitivity), both with regard to genetic abnormalities as well as constitutive in vitro cytokine release, global and stem cell gene expression profiles, epigenetic characteristics and intracellular pathway activation [12,13,14,66,67,68,69,70,71,72]. Thus, even though leukemic stem cells are regarded as responsible for AML relapse [73], biological characteristics associated with relapse risk are also reflected in the total AML cell population. 

Despite the patient heterogeneity, significant overall differences were observed at the proteome and phosphoproteome level for several biological processes between AML cells from RELAPSE and REL_FREE patients, harvested at the time of diagnosis. In particular, proteins involved in ribosome biogenesis and rRNA processing/regulation were more abundant and more strongly phosphorylated in RELAPSE patient cells. This finding of rRNA processing upregulation was supported by the ChIP-seq data analysis from the K562 cell line, where we found several transcriptions factors promoting the transcription of genes for RNA polymerase (CEBPZ) and ribosome/ribosomal biogenesis (PML, AFF1, ETV6 and TRIM28). Deregulated RNA binding proteins and ribosome biogenesis can be drivers of cancer pathogenesis [74,75]. Furthermore, the GSEA analysis revealed enrichment of MYC targets in RELAPSE patients. MYC is frequently overexpressed in AML as well as other leukemic malignancies, and seems to be a crucial transcriptional factor during hematopoiesis [76,77]. MYC is also a regulator of ribosome biogenesis [78], activates RNA pol I for rDNA transcription [79] and induces snoRNA expression [80]. As MYC targets are enriched during relapse, our findings suggest that MYC, together with proteins involved in rRNA processing and ribosome biogenesis, are prognostic biomarkers for relapse and potential therapeutic targets in AML. Several drugs targeting rRNA synthesis, transcription or processing in hematological cancers have already been described [81,82]. 

We observed an increased abundance of V-ATPase subunits in REL_FREE patients. The V-ATPase complex can modulate several intracellular signaling pathways with importance in AML (such as PI3K-mTOR [83,84], NOTCH1 [85] and WNT [86]) by controlling acidification of intracellular compartments. V-ATPase is also important for autophagy [87]. Two previous studies have described an association between low expression at the mRNA level of single autophagic regulators and an adverse prognosis. First, BECN1 is important for the initiation and advance of autophagy, and reduced *BECN1* mRNA levels were associated with an adverse prognosis, FLT3-ITD, monosomal karyotype and high age [88]. Second, decreased *BECN1* mRNA expression is also associated with an adverse prognosis [89]. Thus, our present studies suggest that the expression profile of mediators involved in autophagy is associated with prognosis in human AML, and to the best of our knowledge our present study is the first to describe this for several mediators and at the protein level.

When testing the effect of V-ATPase inhibitor on AML cells, we observed that the RELAPSE derived cells with initially low abundance of V-ATPase subunits responded better to BafA1 V-ATPase inhibitor, thus, a potential relationship between low initial V-ATPase subunit abundance and chemoresistance needs further evaluation. V-ATPase is regarded as a potential therapeutic target in AML [90]. The role of autophagy in AML is complex. Autophagy is often reduced in human AML cells and the loss of key autophagy genes leads to leukemia initiation and progression in mouse models [91]. On the other hand, autophagy seems important for the pro-survival protection of AML cells, and exposure of the leukemic cells to antileukemic drugs (i.e., cytarabine, anthracyclines and sorafenib) seems to activate and increases autophagic flux and thereby allowing them to resist chemotherapy [92,93,94,95]. These different roles suggest that the effect of therapeutic targeting of autophagy in human AML will differ between patients, and this is also consistent with our present data.

Upregulated iron transport and vesicle proteins were enriched in REL_FREE patients. In an independent study, AML patients with high constitutive cytokine release showed increased mRNA expression of proteins involved in intracellular iron homeostasis and molecular trafficking [66]. Interestingly, the overall survival after intensive chemotherapy was significantly higher for high-release compared with low-release AML patients. These results provided confidence and an internal validation of our approach.

Alignment of sequences surrounding phosphorylation sites and KSEA analysis of our full phosphoproteomics dataset showed an enrichment of phosphorylated substrate groups of MAPK, CSK2, CDK, PAK, PRKC and AKT kinases. We additionally observed that RELAPSE cells had higher activity of CDK2 and higher expression, based on immunoblotting assays, compared to REL_FREE cells. As similarly demonstrated previously, KSEA of phosphoproteome data of AML-derived cells from 20 patients had similar enrichment of CSK2, CDK, PAK, AKT, ABL, SRC, MAPK, PRKA and PRKC kinases, relative to normal peripheral blood stem CD34^+^ cells from five healthy donors [45]. Thus, two independent phosphoproteomics studies on different AML patient cohorts have identified similar sets of kinase upregulations in AML. 

The CSK2 inhibitor CX-4945 had a stronger antiproliferative effect for REL_FREE cells than for RELAPSE cells, when acting alone or in combination with CDK inhibitors. Further studies are required to determine whether RELAPSE cells owe their higher CSK2 inhibitor resistance to generally increased robustness or to the lower turnover of CSK2-mediated phosphorylation. Comprehensive KSEA, sequence logos and antibody blotting analyses show that CSK2 and CDKs could be suggested as strong RELAPSE promoters. CSK2, in particular, is a key regulator of several signaling networks and is overexpressed in many hematological cancers [96]. The activity of CDK2 (and potentially also CDK4, CDK6, CDK7 and CDK9) in RELAPSE patients may further increase AML cell proliferation by triggering G1-S-phase transitions [97]. 

Several phosphoproteins were multi-phosphorylated, like BCL11A with several phosphosites upregulated in the RELAPSE group (S205, T208, S608, S625, S630 and S718). BCL11A, a transcription factor associated with the BAF SWI/SNF chromatin remodeling complex, may play a role in leukemogenesis [98] and its expression is associated with adverse outcome in AML [99]. In a previous phosphoproteomics study with AML patients treated with the FLT3 inhibitor quizartinib, increased phosphorylation of BCL11A S630 was associated with non-responsiveness [27]. Taken together, these results suggest that BCL11A phosphorylation contributes to chemoresistance in AML. TP53BP1, another multi-phosphorylated protein in RELAPSE patients, is a double-strand DNA break repair protein that is phosphorylated at multiple sites in response to DNA damage. Moreover, phosphorylation at S1219 by ATM kinase seems to be a response to ionizing radiation [100]. The S523Q motif might also serve as substrate for ATM kinase and being part of the RIF1, a non-homologous end joining-mediated repair protein, recruitment process to DNA break sites [101]. 

The stimulatory T190 and T191 phosphosites in the activation loop of NEK7 showed increased phosphorylation in the REL_FREE patients. NEK7 seems to be involved in the recruitment of centrosomal pericentriolar proteins that are necessary for centriole duplication and spindle pole formation during mitosis [102]. Chromosomal lagging, micronuclei formation, cytokinesis failure and tetraploidy/aneuploidy were observed in NEK7 deficient mouse embryonic fibroblasts [103]. Therefore, deregulation of NEK7 activity in cell division processes might contribute to aberrant mitosis and induction of the relapse status [104]. Furthermore, we found two phosphosites, S372 and S389, in the ribosomal S6 kinase RPS6KA1 isoform 2 (Uniprot identifier: Q15418-2), upregulated in REL_FREE patients. It has been reported that phosphorylation at S372 and S389, possibly by PRKC activators, switched on the RPS6KA1 N-terminal kinase domain required for phosphorylation of its substrates [105]. The role of ribosomal S6 kinases in cancer is not well understood and seems to vary by cancer type. While some isoforms of ribosomal S6 kinases can promote cell motility and invasion processes by altering transcription and integrin activity, others depress them by interacting with the actin cytoskeleton [106]. The fact that the phosphosites of RPS6KA1 might be phosphorylated by NEK7 [104], and not by MTOR, might explain the benefits of higher phosphorylation of membrane, actin filament and cell-cell adherens junction proteins in the REL_FREE group. Our findings encourage functional studies to understand the role of NEK7, PRKC and RPS6KA1 kinases in the cytoskeleton of AML cells. 

Finally, although the time intervals to define early or late relapse are debatable [41,42], we subclassified the 26 RELAPSE into 14 EARLY RELAPSE and 12 LATE RELAPSE patients, after setting up 12 months from diagnosis to relapse as the mark time to differentiate the two groups. Our results showed that the rRNA processing and V-ATPase clusters (identified when all the 26 RELAPSE patients were included) mainly described the proteome landscape at diagnosis for both the EARLY RELAPSE vs. REL_FREE and LATE RELAPSE vs. REL_FREE subsets. Thus, the AML proteome at diagnosis for patients with either an early or late relapse does not seem to change dramatically. In contrast, the different AML phosphoproteome landscapes at diagnosis described in the EARLY RELAPSE vs. REL_FREE and LATE RELAPSE vs. REL_FREE subsets appear to be influenced by the relapse-free time of RELAPSE patients. Our results, considering all the 26 RELAPSE patients, are valuable for therapy decisions at diagnosis. Nonetheless, further studies with diagnostic and relapsed samples at early and late relapsing episodes might better describe the role of different AML relapse determinants.

## 4. Materials and Methods

### 4.1. AML Patients and Sample Collection

Written informed consent was obtained from all patients in accordance with the Declaration of Helsinki, and the use of human leukemia cells for the present study was approved by the Regional Ethics Committee (REK III Vest 2013-634). Primary AML cells were collected from the peripheral blood of 41 patients with relatively high levels of circulating leukemia blasts (>80% of circulating leukocytes) at the first time of diagnosis, prior to any antileukemic therapy. The peripheral blood leukocyte counts are given in Table S1. Six of the patients had leukocyte counts exceeding 100 × 10^9^/L and six additional patients had leukocyte counts between 50–100 × 10^9^/L, whereas most patients had leukocyte counts below 50 × 10^9^/L. Peripheral blood samples were collected on ACD tubes and highly enriched AML cell populations (generally >95%) could then be prepared by density gradient separation alone (Lymphoprep, Axis-Shield, specific density 1.077) [107]. The gradient separation and cryopreservation were done immediately after sampling, by using the same standardized method for all patients [31]. Cells were stored in liquid nitrogen until used in the experiments. Flow cytometric analyses showed that the main contaminating cell population, as expected, was small lymphocytes.

Our department is responsible for the diagnosis and initiation of intensive antileukemic treatment for all AML patients in a defined geographical area. The patients in the present study represent all patients within this area and time period who (i) either completed their planned intensive induction and consolidation therapy or died from primary resistant AML/early chemoresistant relapse during treatment, (ii) had a high percentage of circulation AML cells in peripheral blood, and (iii) had an observation time of at least five years. Patients dying from or ending the planned antileukemic therapy due to severe treatment-related toxicity were not included in our present study. Our patients could therefore be classified into two contrasting groups: 15 patients were classified as relapse-free based on the long follow-up period (i.e., REL_FREE patients) and 26 patients as chemoresistant/relapse patients (i.e., RELAPSE patients), including all patients with primary resistant, early relapse during initial chemotherapy or late relapse after completed consolidation therapy. Some of the patients in this last group later received allogeneic stem cell transplantation. 

We compared AML cells derived at the first time of diagnosis prior to any antileukemic treatment (see Supplementary methods) for the two contrasting patient groups, and the samples did not differ in storage time or the peripheral blood leukocyte counts of the patients at the time of first diagnosis/sampling. Additional and more detailed patient information is given in Figure 1a,b, Table 1 and Table S1, including the results from karyotyping and mutational analysis of 54 genes frequently mutated in AML. The method for mutational analyses has been described previously [108]. Mutations were classified according to their biological function [34].

Nine additional AML patients were included in validation studies and are described in detail in Table S2. 

### 4.2. AML Super-SILAC Mix

An AML spike-in reference was generated by combining Arg6- and Lys8-labeled protein lysates from five heterogeneous AML-derived cell lines, according to the super-SILAC mix approach [30,109]. The spike-in reference was added to each patient sample in a 1:2 ratio (w:w; super-SILAC mix:AML patient sample) for SILAC-based quantitation.

### 4.3. Patient Sample Preparation for Proteomic and Phosphoproteomic Analysis

Sample preparation of patient cell lysate in 4% sodium dodecyl sulfate (SDS)/0.1 M Tris-HCl (pH 7.6) and immobilized metal affinity chromatography (IMAC) has been described earlier [32]. Briefly, 20 µg of each patient lysate was prepared both as 1) a label-free sample and 2) mixed with 10 µg of the super-SILAC mix for proteomic analyses, and processed according to the FASP protocol (Figure 1c) [32,110]. Only the super-SILAC spiked peptide samples were fractionated using styrenedivinylbenzene-reversed phase sulfonate (SDB-RPS) plugs (Empore, 3M) [111]. The phosphoproteomics samples (64–1121 µg range) were mixed with the super-SILAC mix at the ratio described before, FASP processed and enriched for phosphopeptides using the IMAC procedure.

### 4.4. Nanoflow LC-MS/MS

Peptide sample preparation prior to LC-MS/MS and settings of the LC-MS/MS runs on a Q Exactive HF Orbitrap mass spectrometer coupled to an Ultimate 3000 Rapid Separation LC system (Thermo Scientific, Waltham, MA, USA) were conducted as described earlier for global proteomics [112] and phosphoproteomics [32], and detailed in Supplementary Methods.

### 4.5. Data and Bioinformatics Analysis

LC-MS/MS raw files from RELAPSE and REL_FREE samples were processed with MaxQuant software version 1.5.2.8 (see Supplementary methods) [113,114]. The spectra were searched against the concatenated forward and reversed-decoy Swiss-Prot Homo sapiens database version 2018_02, using the Andromeda search engine [115]. The LC-MS/MS raw files and MaxQuant output files have been deposited to the ProteomeXchange consortium via the PRIDE partner repository [116,117] with dataset identifier PXD014997. The Perseus 1.6.1.1 platform was used to analyze and visualize protein groups and phosphosites [118]. MaxQuant-normalized SILAC ratios were inverted, log_2_ transformed and normalized again, using width adjustment. Proteins and phosphosites (localization probability > 0.75) with at least five individual SILAC ratios in each patient group were selected for two-sample unequal variance *t*-test and *Z*-statistics [119] to find significantly different FC for proteins and phosphosites between the RELAPSE and REL_FREE groups. Hierarchical clustering of significantly differential proteins and phosphosites was done with Perseus using the Pearson correlation function and complete linkage. GO analysis was performed using a GO tool [120]. The most significantly over-represented GO terms with FDR < 0.05 were displayed in bar plots in Prism8 (GraphPad). Venn diagrams were made with Venny 2.1 (*Oliveros, J.C.,* http://bioinfogp.cnb.csic.es/tools/venny/index.html) or BioVenn [121]. GSEA (http://software.broadinstitute.org/gsea/index.jsp) was performed against the Hallmark gene set collection of the Molecular Signatures Database v6.2 [122]. The amino acid distribution surrounding the phosphosites was analyzed using iceLogo (*p* = 0.05), with the sequence windows obtained in the MaxQuant-generated phosphosite output file [43]. Unregulated phosphosites were used as a reference set. Sequence logo analyses from a small number of phosphopeptide sequences were generated with WebLogo [46]. Kinase activity estimates were inferred by the KSEA App. [44,45]. Regulated and unregulated phosphosites were analyzed with the PhosphoSitePlus [123] and NetworKin [124] databases, using a substrate count and a NetworKin score cutoff of 5. Significantly regulated kinases with Benjamini–Hochberg adjusted *p* < 0.05 were showed in bar plots. Kinase activation loop analysis was performed with the tools for phosphoproteomics data analysis at http://phomics.jensenlab.org. PPI networks were obtained by using the STRING database version 10.5, with interactions derived from experiments and databases at a high confidence score of 0.7 [125]. Networks were visualized using the Cytoscape platform version 3.3.0 [126]. The ClusterONE plugin was used to identify protein groups of high cohesiveness [127].

### 4.6. Enrichment Analysis of Transcription Proteins Binding Sites

Putative binding sites of transcription proteins were found by analyzing public available ChIP-seq data on K562 cell line (derived from a patient with blast phase chronic myeloid leukemia; an undifferentiated myeloid cell line also expressing certain erythroid markers [128,129]) at the ENCODE project [130]. ENCODE accession IDs of files are provided in Table S3. Sequencing reads were downloaded and aligned to human genome hg19 using Bowtie 2.2.9 [131]. Aligned reads were subjected to peak calling using MACS and annotated using Homer following methods published previously [132,133,134,135,136]. Peak information of the putative targets was analyzed by the BETA algorithm in Cistrome analysis pipeline (http://cistrome.org/ap/root) [137]. A GO enrichment analysis of putative target genes was carried out using DAVID with default parameters [138,139]. Significantly over-represented GO terms with Benjamini–Hochberg adjusted *p* < 0.05 were displayed in bar plots.

### 4.7. Cell Proliferation Assay

The cytokine-dependent in vitro proliferation of enriched AML cells was analyzed by a 3H-thymidine incorporation assay [56]. Cryopreserved primary cells were thawed in a water bath set at 37 °C and diluted 1:1 with RPMI, containing 2 mM L-glutamine and penstrap. The cells were incubated at room temperature for 5 min and extra RPMI solution (same volume of the original cells) was added. The cells were centrifuged at 250× *g* at room temperature for 8 min and the pellet was resuspended in 4.5 mL of the RPMI solution. After counting the cells with trypan blue, the AML cells were seeded in flat-bottomed 96-well microtiter plates (VWR^®^, Lutterworth, UK), with a seeding density of 50,000 cells/well (200 μL medium/well). The culture medium was Stem Span SFEM™ (Stem Cell Technologies, Vancouver, Canada), supplemented with granulocyte-macrophage stimulating factor (GM-CSF), FLT3 ligand (FLT3-L) and stem cell factor (SCF); all cytokines were purchased from PeproTech (Rocky Hill, NJ, USA) and used at a final concentration of 20 ng/mL. The cells were treated with 0 (control), 1, 5 and 10 nM BafA1 (*Streptomyces griseus*—CAS 88899-55-2—Calbiochem, now Sigma-Aldrich, St. Louis, MO, USA) and the kinase inhibitors (Selleckchem, Houston, TX, USA) at concentrations indicated in Table S5. All the inhibitors used in this study are well characterized and more detailed information including clinical trials is available in the PubChem database [140]. After six days of culture, 37 kBq of ^3^H-thymidine (Perkin Elmer, Waltham, MA, USA) was added to each well and the cells were further incubated for 18–20 h, before being harvested and nuclear radioactivity being measured. For cell viability assay of cells treated with 10 nM BafA1 and control, primary cells were cultured for 48 h in flat-bottomed 24-well culture plates (VWR^®^) (1 × 10^6^ cells/mL, 1 mL/well), using the same medium as described above. The cells were labeled with propidium iodide and fluorescein isothiocyanate-conjugated Annexin V (Tau Technologies BV, Kattendijke, The Netherlands), prior to flow cytometry analysis. The percentage of viable, apoptotic and necrotic cells was determined. The median of triplicate determinations was used in all analyses. A Mann–Whitney U test (SPSS Statistics v.25, IBM, Armonk, NY, USA) was employed for the analysis of differences between different groups and Wilcoxon’s matched-pair signed rank test (SPSS Statistics v.25) for the analysis of differences between paired samples.

### 4.8. Western Blots

Western blots for three RELAPSE and three REL_FREE patient cells used in the cell proliferation assay and incubated with kinase inhibitors at 37 °C for 15 min were performed. Control and treated patient primary cells were centrifuged at 270× *g* at 4 °C for 5 min and washed with a solution of NaCl (9 mg/mL) on ice. After the centrifugation step, 100 µL of 4% SDS/0.1 M Tris-HCl (pH 7.6) were added to the cell pellets. The samples were heated at 95 °C for 7 min and kept in the freezer. Twenty-five µg of each sample was loaded on a NuPAGE 4–12% Bis-Tris protein gel (ThemoFisher Scientific) and transferred onto a nitrocellulose membrane (Amersham Protran, GE Healthcare Life Sciences, Chicago, IL, USA) in an XCell II Blot Module (ThermoFisher Scientific). Antibodies were purchased from Cell Signaling Technology (Danvers, MA, USA) and used according to the manufacturer’s guidelines. Chemiluminescence was measured on a LAS-3000 imager (Fujifilm, Tokyo, Japan). Band intensities for each protein were determined by densitometry software Image J (Table S6) [141]. Band intensities of a protein spotted at approximately 17 kDa on Ponceau-stained membranes were used for normalization (Figure S13a, Table S6). Statistical analysis was performed using unpaired *t*-test.

## 5. Conclusions

This study describes the proteomics profiles of patient-derived AML cells collected at the first time of diagnosis that differ between patients that are long-term relapse free survivors and patients that relapse. The relapse-associated group displayed decreased expression of V-ATPases and the increased activity of CDKs and CSK2. Furthermore, our results suggest a subset of transcriptional and metabolic regulators that could be considered as predictors of prognosis and therapeutic targets in AML.

## Figures and Tables

**Table 1:** Characteristics of the 41 AML patients used in the study at the first time of diagnosis.

Characteristic	REL_FREE	RELAPSE
Age average (range) in years	49.5 (36–65)	50.5 (18–68)
Number of patients	15	26
**FAB classification**		
M0-M1	1	11
M2	0	2
M4-M5	14	12
uncertain	0	1
***FLT3***		
WT	14	14
ITD	1	8
*ND*	0	4
***NPM1***		
WT	6	16
Ins	8	7
*ND*	1	3

FAB: French-American-British; WT: wild type; ITD: internal tandem duplication; Ins: a 4 bp-insertion/duplication; *ND* indicates not determined

## Supplementary Materials

The following are available online at https://www.mdpi.com/2072-6694/12/3/709/s1, Supplementary methods with more explanations on the treatment of AML patients, LC-MS/MS analysis of the AML proteome and phosphoproteome, and MaxQuant analysis of SILAC-labeled and label-free AML samples. Supplementary data showing the comparison between the proteomics analyses of the datasets produced using the super-SILAC mix and label-free quantification methods. Figure S1: The number of quantified proteins per patient sample is similar with label-free and super-SILAC mix based quantification. Figure S2: The FCs of RELAPSE vs. REL_FREE proteins correlate in the super-SILAC mix and label-free datasets. Figure S3: Clustering performance of 150 proteins validated by label-free proteomics. Figure S4: GSEA analysis. Figure S5: Comparison between the differentially expressed proteins found in the EARLY RELAPSE-REL_FREE and LATE RELAPSE-REL_FREE subcohorts. Figure S6: IceLogos of the 31 amino acid sequence windows surrounding phosphorylation sites (location marked with an arrow). Figure S7: Sequence logo analysis of the 31 amino acid sequence windows surrounding phosphorylation sites (located on position 16 on the X axis) of ClusterOne protein networks showed in Figure 3e on the main text. Figure S8: Significant GO molecular function terms and KEGG pathways of target genes predicted from analysis of ChIP-seq data of transcription proteins. Figure S9: Phosphoproteins with differentially regulated phosphorylation sites in the RELAPSE vs. REL_FREE cohort dataset with unaltered overall expression. Figure S10: Overlap of regulated proteins and phosphoproteins in the RELAPSE vs. REL_FREE cohort study. Figure S11: Comparison between the differentially regulated phosphorylation sites found in the EARLY RELAPSE-REL_FREE and LATE RELAPSE-REL_FREE subcohorts. Figure S12: V-ATPase inhibitor assays. Figure S13: Ponceau-stained membranes and Western blots. Table S1: Overview of the 41 AML patients used in this study. Table S2: Overview of the nine AML patients external to the study cohort used for cell proliferation and Western blot assays. Table S3: List of 14 transcription-related proteins and/or phosphoproteins with higher abundance/phosphorylation in the RELAPSE group subjected to ChIP analysis to determine binding sites of target genes. Table S4: List of overlapping regulated proteins and phosphoproteins in the 26 RELAPSE and 15 REL_FREE patient cohort. Table S5: Cell proliferation assays using kinase inhibitors. Table S6: Densitometry intensities and normalized intensities of Western blot bands. Supplementary References. Supplementary file 1: Proteomics data RELAPSE vs. REL_FREE cohort. Supplementary file 2: Supp information to Figure 2c. Supplementary file 3: Phosphoproteomics data RELAPSE vs. REL_FREE cohort. Supplementary file 4: Kinase substrates.
