# Supplementary material for: Proteome and Phosphoproteome Changes Associated with Prognosis in Acute Myeloid Leukemia
Source: Cancers (Basel). 2020 Mar 17;12(3):709. doi: 10.3390/cancers12030709 (PMC7140113; doi:10.3390/cancers12030709)
Supplement: Supplementary file 1 [file cancers-12-00709-s001.zip › cancers-724561-SI.pdf]

cancers

Article

Proteome and Phosphoproteome Changes Associated with Prognosis in Acute Myeloid Leukemia

Elise Aasebø, Frode S. Berven, Sushma Bartaula-Brevik, Tomasz Stokowy, Randi Hovland, Marc Vaudel, Stein Ove Døskeland, Emmet McCormack, Tanveer S. Batth, Jesper V. Olsen, Øystein Bruserud, Frode Selheim and Maria Hernandez-Valladares

Supplementary Methods

Treatments of AML Patients

All the patients were treated with intensive induction chemotherapy (seven days of nucleoside analog cytarabine and three days of an anthracycline antibiotic, daunorubicin or idarubicin) and followed up during and after treatment. The patients were further treated with consolidation therapy, which consisted of either high-dose (3 g/m^2) monotherapy [1] or intermediate-dose (1 g/m^2) cytarabine in combination with one or two additional cytotoxic drugs.

LC-MS/MS Analysis of the AML Proteome and Phosphoproteome

Dried peptides were dissolved in 2% ACN/0.5 % FA. Peptides (approximately 1 µg for proteomic and phosphoproteomic analysis) were pre-concentrated on a 2 cm × 75 µm ID Acclaim PepMap 100 trapping column and separated on a 50 cm × 75 µm ID EASY-spray PepMap RSLC analytical column (Thermo Scientific, Waltham, MA, USA.). Samples were analyzed on a Q Exactive HF Orbitrap mass spectrometer equipped with an Easy-Spray (Thermo Scientific) and coupled to an Ultimate 3000 Rapid Separation LC system (Thermo Scientific). For the proteomic samples, the peptides were eluted during a 195 min binary gradient with solvent A (0.1% FA) and solvent B (0.1% FA/ACN). The gradient started at 5% B from 0–5 min and increased to 8% B from 5–5.5 min, then to 24% B from 5.5–115 min, to 35% B from 115–140 min, and to 90% B from 140–155 min. Hold at 90% from 155-170 min, then ramped to 5% B from 170–195 min. The first fraction of the three SDB-RPS fractions was eluted with a similar, but slower gradient, with increase from 5–7% B from 5–5.5 min, and from 7–22% B from 5.5–115 min. The Q Exactive HF mass spectrometer was operated in data dependent acquisition (DDA) mode. Full MS scans (scan range 375–1500 m/z) were acquired in profile mode with a resolution *R* = 120 000, a target value of 3×10^6 and maximum injection time of 100 ms. MS/MS scans were acquired in centroid mode for the top 12 precursors with intensity threshold >5×10^4 (5.5% underfill ratio). The target ion was set to 1×10^5 with a maximum injection time of 110 ms and a resolution *R* = 30 000. The normalized collision energy was 28, the isolation window was 1.6 m/z with 0.3 m/z offset, and the dynamic exclusion lasted for 25 s. For the SILAC samples the lock mass at 445.12003 m/z was enabled. For the phosphoproteomic sample, the phosphopeptides were also eluted during a 195 min binary gradient with the solvents described above. The gradient started at 5% B from 0–5 min and increased to 7% B from 5–6 min, then to 12% B from 6-60 min, to 38% B from 60–145 min, to 90% B from 145–150 min. Hold at 90% from 150–170 min, then ramped to 5% B from 170–175 min and hold at 5% until 195 min. The Q Exactive HF mass spectrometer was also operated in DDA mode with an adjusted acquisition method published by others [2]. Full MS scans (scan range 375–1500 m/z) were acquired in profile mode with a resolution *R* = 60 000, a target value of 3×10^6 and maximum fill time of 15 ms. MS/MS scans were acquired in profile mode for the top 10 precursors with intensity threshold 1×10^5 (11% underfill ratio). The target ion was set to 1×10^5 with a maximum injection time of 110 ms and a resolution *R* = 60 000. The normalized collision energy was 28, the isolation window was 1.2 m/z and the dynamic exclusion lasted for 30 s.

The label-free and super-SILAC proteomic and phosphoproteomic samples were analyzed as three separate experiments in a controlled randomized order (i.e., samples from each patient group were distributed more or less equally over the analysis sequence) with LC-MS quality controls (LC-

MS-QC) run approximately every 10 patient samples. As LC-MS-QC for the label-free experiment, we used a pool of all samples included in the analysis, while a HeLa protein digest and a phosphopeptide pool made in our laboratory were used as LC-MS-QC for the super-SILAC spiked experiments.

MaxQuant Analysis of SILAC-Labeled and Label-Free AML Samples

MaxQuant parameters were set up for SILAC-labeled samples as follows. Cysteine carbamidomethylation was used as a fixed modification; methionine oxidation, protein N-terminal acetylation, Gln->pyro-Glu and serine/threonine/tyrosine phosphorylation (only for phosphoproteome parameter group) as variable modifications. Trypsin was used as digestion protease. The false discovery rate was set at 0.01 for phosphosites, peptides and proteins. The minimum peptide length allowed was six amino acids. The match-between-runs and re-quantify options were enabled.

All patient lysates were processed and analyzed as label-free samples in a different MaxQuant run and used to support the quantitative results from the super-SILAC data. We used the MaxLFQ quantification [3] for relative label-free quantification, with LFQ count set to 1, where an optimized MaxLFQ algorithm is used to normalize each protein intensity based on peptide ratios measured in all pair-wise comparisons of the entire sample batch. The algorithm performance is optimal for samples of high similarity analyzed on the same LC-gradient (private communication with the developers).

Only protein groups with identical Uniprot accession numbers were matched to the super-SILAC data, hence, we had corresponding label-free quantitative data for 4041 proteins quantified in the super-SILAC dataset.

Supplementary Data

To compare the results from the super-SILAC mix and label-free datasets, we initially compared the number of quantified proteins per patient sample. The quantified protein numbers in the super-SILAC experiment ranged from 4350 to 5248 proteins per patient, with an average of 4944 proteins. When comparing to the label-free analysis, we observed that analysis of the same patient sample by two quantification methods produced similar numbers of quantified proteins (Figure S1). The average number of proteins quantified per patient sample in the label-free analysis was 4857 proteins; however, the number per patient varied more in this dataset, ranging from 3573 to 5222 proteins. Thus, we had more equal protein number measurements in the super-SILAC dataset, possibly due to peptide fractionation, which increases the detection of low abundant peptides. We further assessed the similarity between super-SILAC and label-free measurements by correlating the fold changes (FC) between the RELAPSE and REL_FREE groups (Pearson $R = 0.72$) (Figure S2). The good FC correlation indicated high similarity between the two different proteomic quantitation approaches. The SILAC data is generally considered as one of the most accurate quantification methods in large-scale proteomics because the internal standard is added to the sample before sample preparation [4]. Finally, the expression difference of 150 of the 351 significantly regulated proteins in the super-SILAC experiment, i.e., 87 increased and 63 decreased, was supported by the label-free dataset ($p < 0.05$). Hierarchical clustering using the super-SILAC and label-free protein ratios/abundances of these 150 regulated proteins produced comparable clustering of the AML patients (Figure S3).

Supplementary Figures

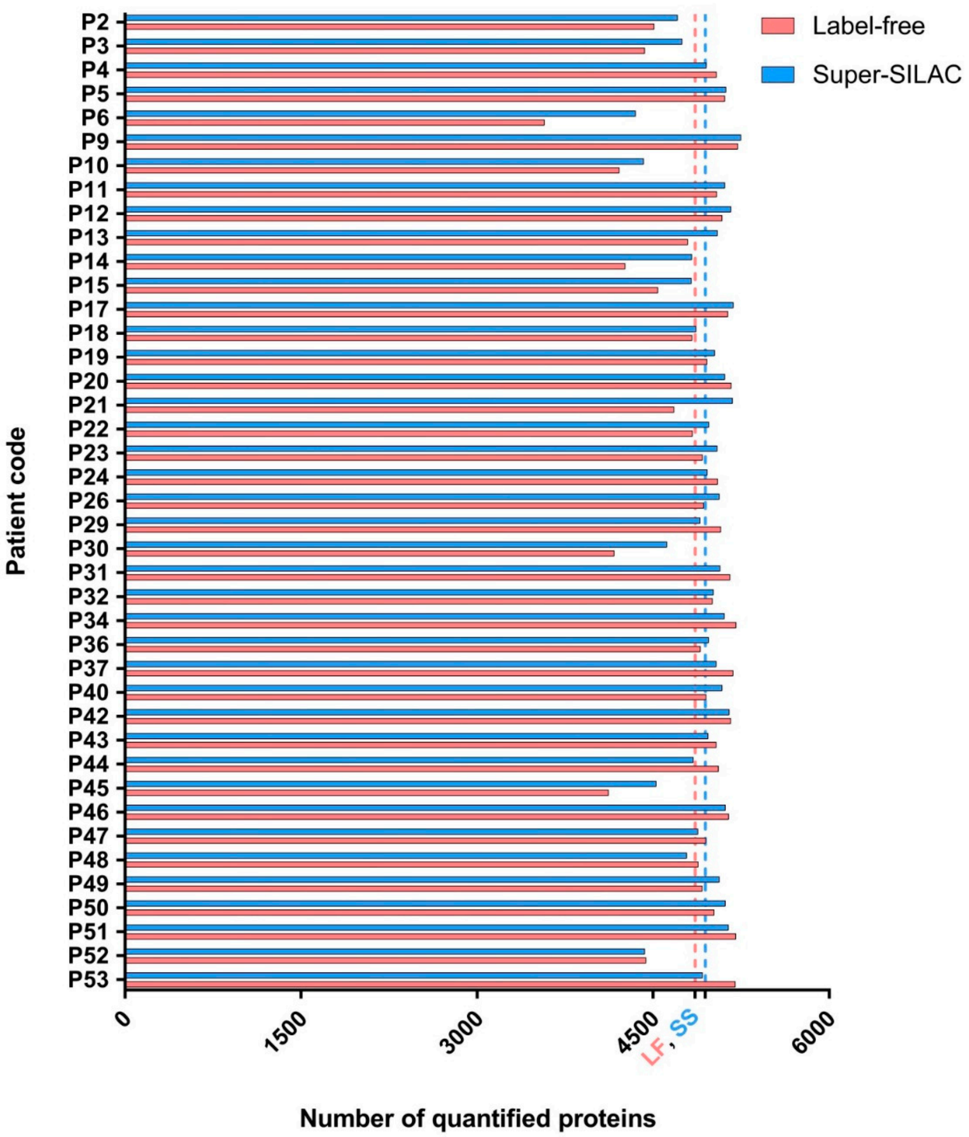

Figure S1. The number of quantified proteins per patient sample is similar with label-free and super-SILAC based quantification. The average numbers of quantified proteins per approach is indicated by the dotted line, i.e., 4857 proteins with label-free and 4944 with super-SILAC based quantification. Patient samples with few quantified proteins in one experiment, typically have few quantified proteins in the other experiment as well (e.g., P6 and P45).

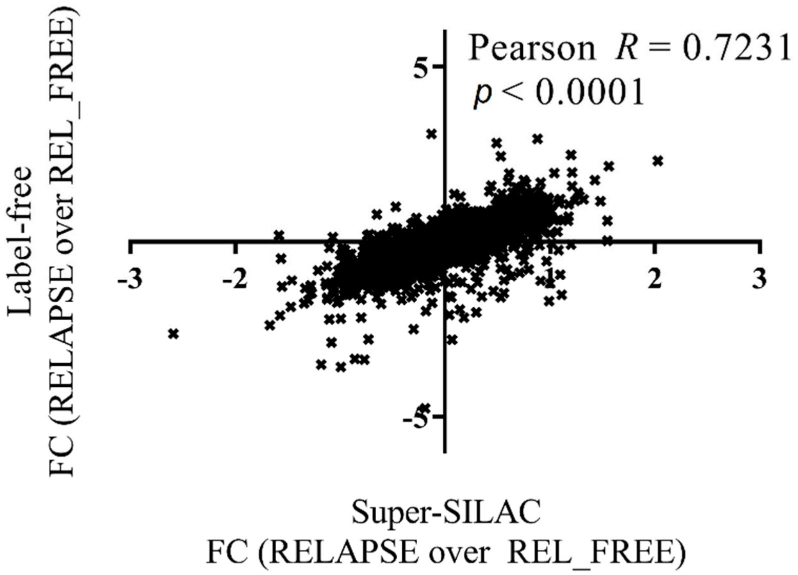

Figure S2. The FCs of RELAPSE *vs* REL_FREE proteins correlate in the super-SILAC and label-free datasets. Pearson *R* value and correlation plot of protein FCs obtained from label-free vs. super-SILAC experiments comparing 26 RELAPSE and 15 REL_FREE patient samples.

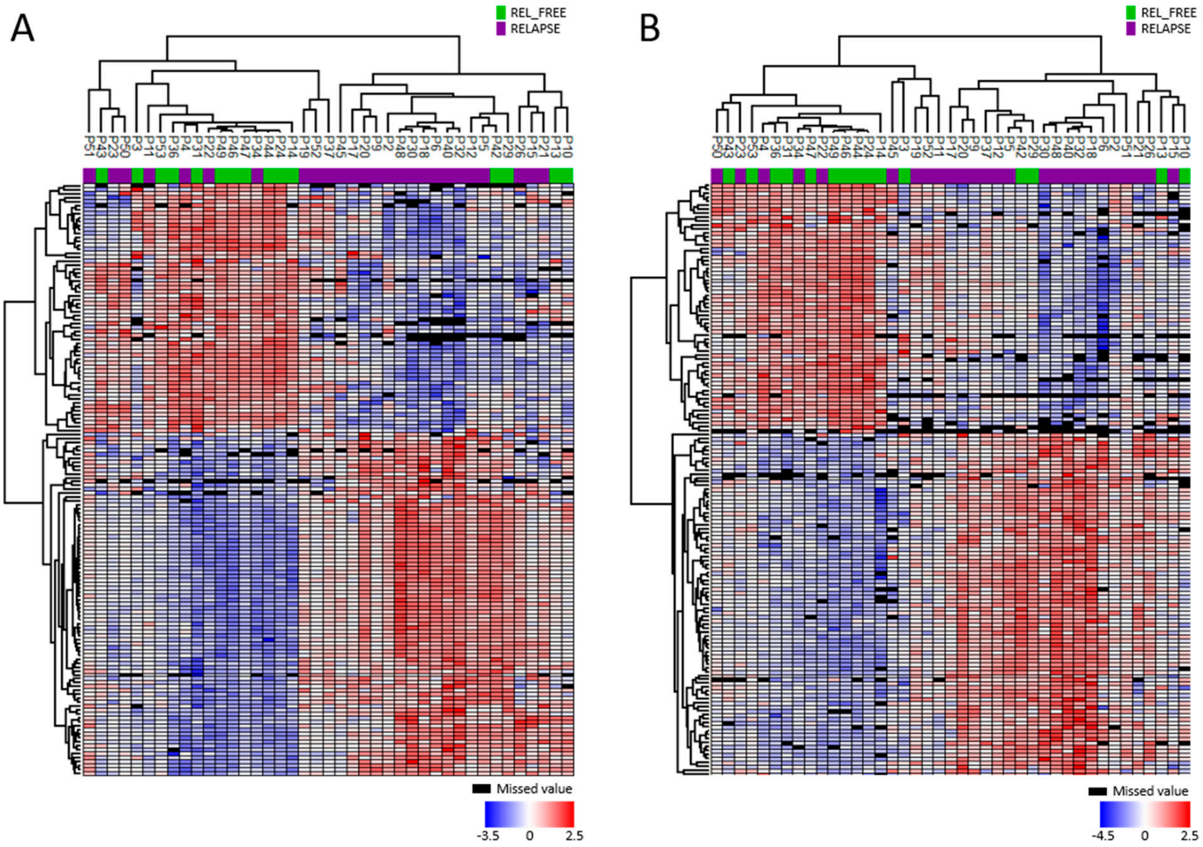

Figure S3. Clustering performance of 150 proteins validated by label-free proteomics. Of the 351 regulated proteins in the super-SILAC dataset, were 150 proteins regulated in the label-free dataset. Hierarchical clustering of the (**a**) the 150 proteins in the super-SILAC dataset and (**b**) the same 150 proteins using label-free data showed similar clustering performance.

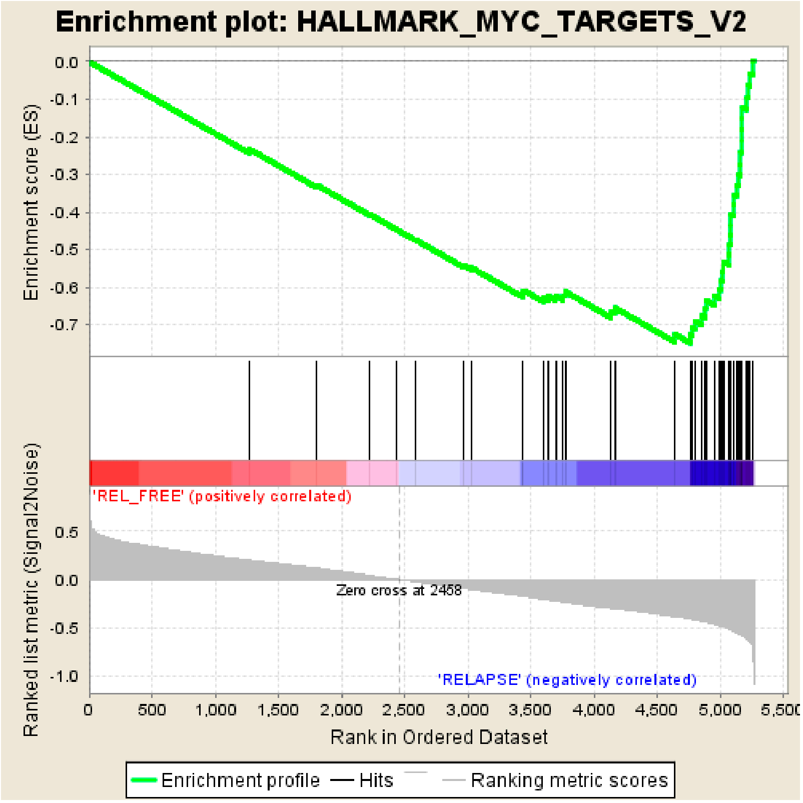

Figure S4. GSEA analysis. GSEA using the 5309 proteins in the RELAPSE vs. REL_FREE cohort study as input data against the Hallmark gene set collection. HALLMARK_MYC_TARGETS_V2 was significantly enriched in the RELAPSE group (Nominal $p < 0.01$, FDR $q = 0.0461$, Normalized enrichment score (NES) = −1.8378). The gene set comprises the 58 following genes: AIMP2, BYSL, CBX3, CDK4 *, DCTPP1, DDX18, DUSP2*, EXOSC5, FARSA, GNL3, GRWD1, HK2, HSPD1, HSPE1, IMP4, IPO4, LAS1L, MAP3K6 *, MCM4, MCM5, MPHOSPH10, MRTO4, MYBBP1A, MYC *, NDUFAF4, NIP7, NOC4L, NOLC1, NOP16, NOP2, NOP56, NPM1, PA2G4, PES1, PHB, PLK1 *, PLK4 *, PPAN, PPRC1 *, PRMT3, PUS1, RABEPK, RCL1, RRP12, RRP9, SLC19A1 *, SLC29A2 *, SORD, SRM, SUPV3L1, TBRG4, TCOF1, TFB2M, TMEM97 *, UNG *, UTP20, WDR43, WDR74 (underlined gene names indicate the 30 proteins contributing the leading edge subset; * indicates 11 proteins that were not quantified in our data).

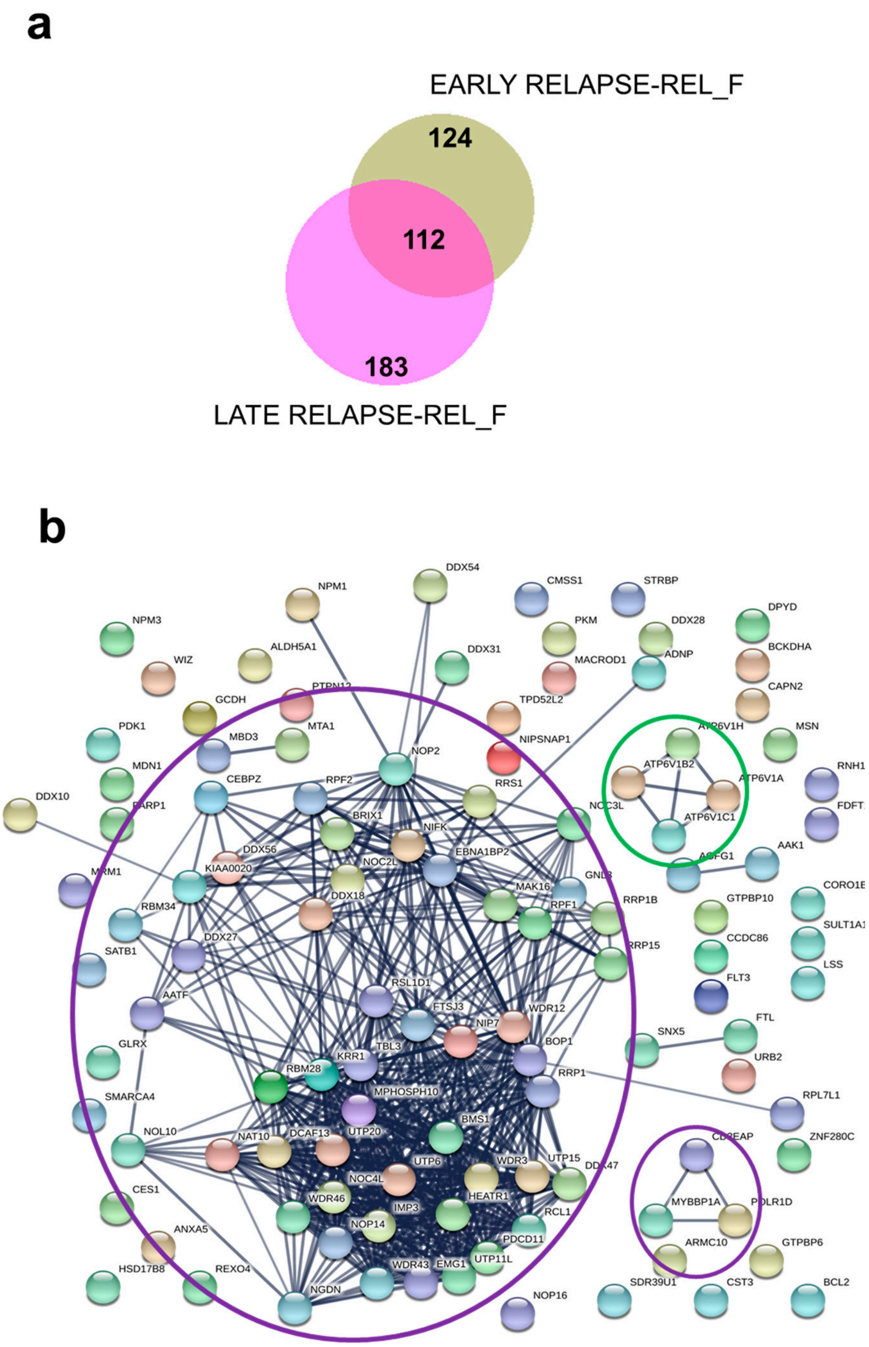

Figure S5. Comparison between the differentially expressed proteins found in the EARLY RELAPSE vs. REL_FREE (EARLY RELAPSE-REL_F in the plot) and LATE RELAPSE vs. REL_FREE (LATE RELAPSE-REL_F in the plot) subcohorts. (**a**) Overlap of the regulated proteins found in the two subcohorts illustrated with a Venn diagram. (**b**) PPI network performed with STRING of the 112 overlapped regulated proteins. Protein networks characterized in Figure 2c of the main text are highlighted here with a colored oval (purple for protein networks with higher abundance in RELAPSE and green with higher abundance in REL_FREE). Black edges mean confidence at 0.7 as interaction score.

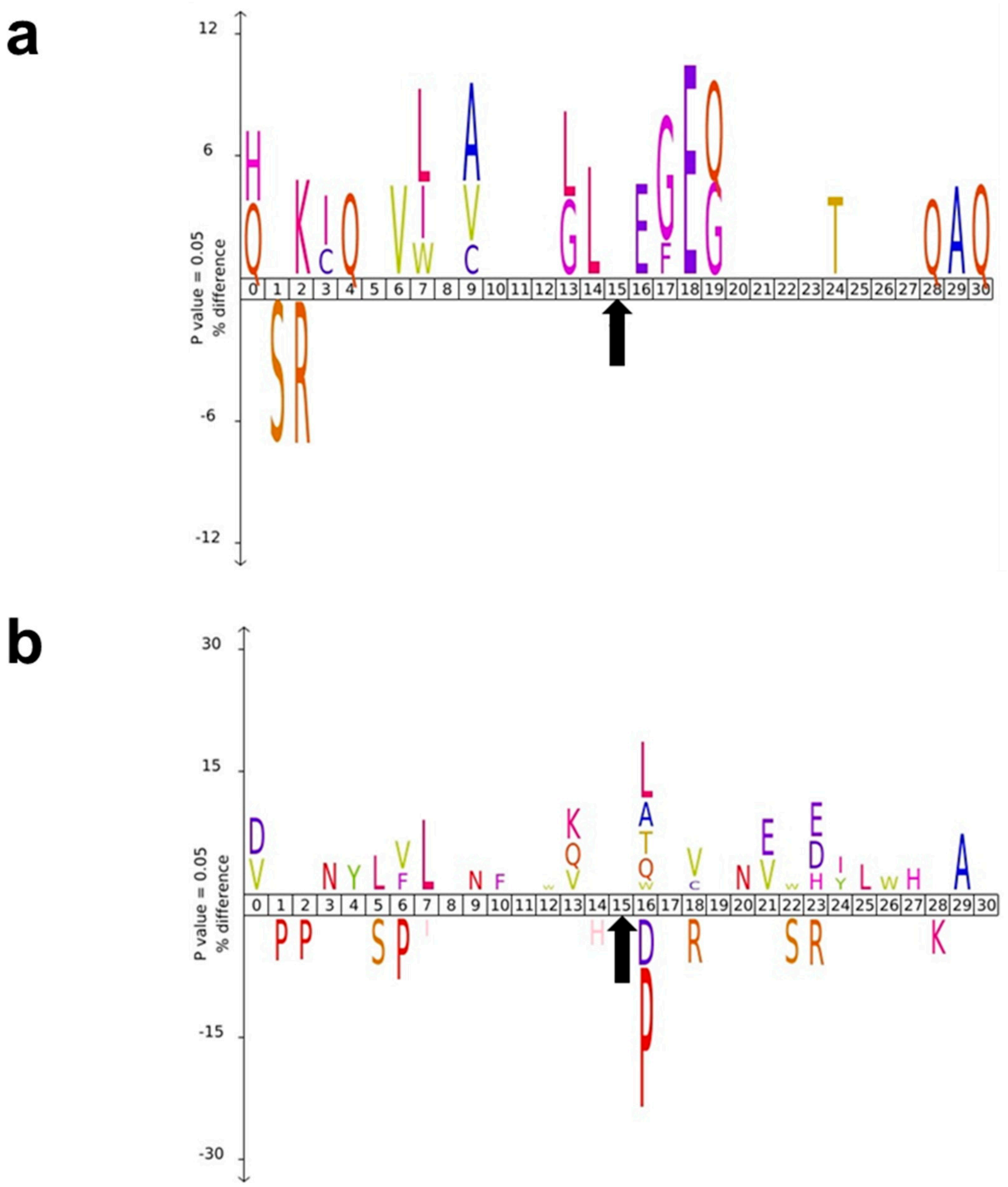

Figure S6. IceLogos of the 31 amino acid sequence windows surrounding phosphorylation sites (location marked with an arrow). (**a**) Alignment of the sequence windows of 138 phosphosites upregulated in the RELAPSE group using the sequence windows from unregulated phosphosites as the reference set. (**b**) Alignment of the sequence windows of 136 phosphosites upregulated in the REL_FREE group using the sequence windows from unregulated phosphosites as the reference set.

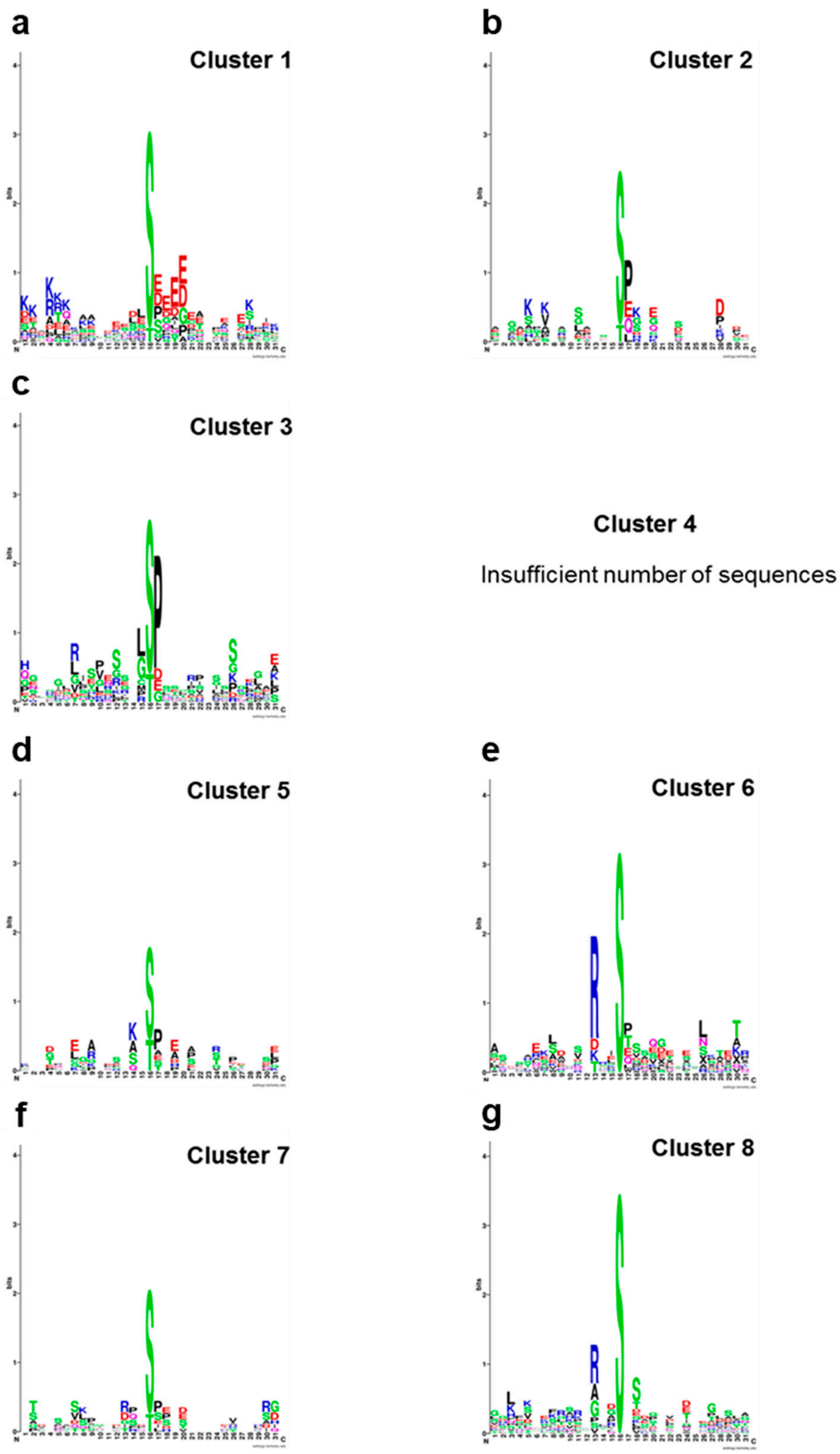

Figure S7. Sequence logo analysis of the 31 amino acid sequence windows surrounding phosphorylation sites (located on position 16 on the X axis) of ClusterOne protein networks showed in Figure 3e on the main text.

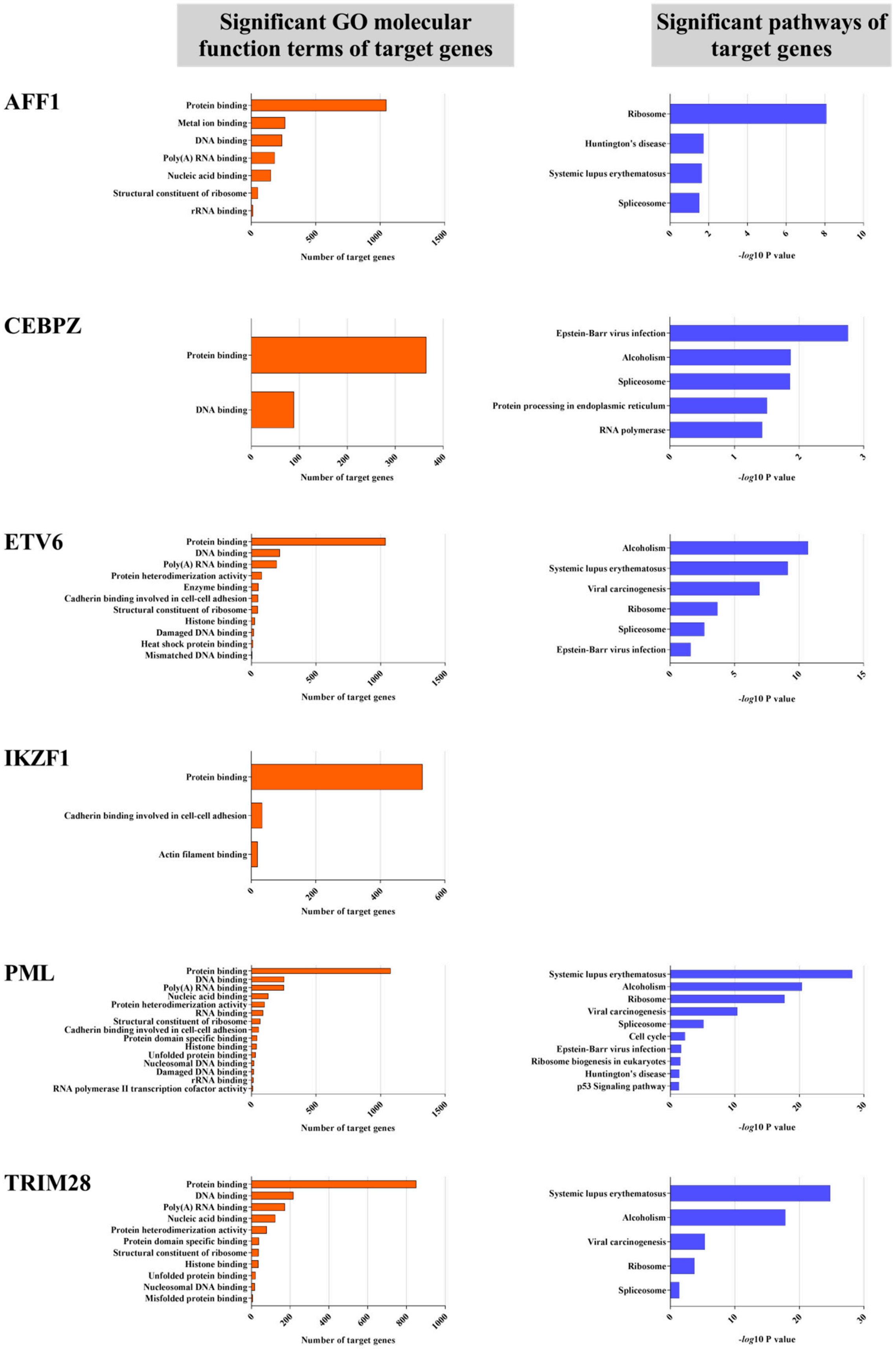

Figure S8. Significant GO molecular function terms and KEGG pathways of target genes predicted from analysis of ChIP-seq data of transcription proteins. All GO terms and pathways were

significantly over-represented with Benjamini-Hochberg adjusted p values < 0.05. Number of target genes and enrichment significance are shown for GO molecular function and pathways terms, respectively. Huntington's disease pathway involves gene transcription, vesicular transport, oxidative phosphorylation and apoptosis elements. Systematic lupus erythematosus involves T-cell receptor signaling, nucleosome, complement and coagulation cascade, and leukocyte transendothelial migration elements. Alcoholism involves PKA and MAPK signaling elements. Epstein Barr virus infection involves B cell receptor signaling, proteasome, basal transcription factors and MAPK signaling elements.

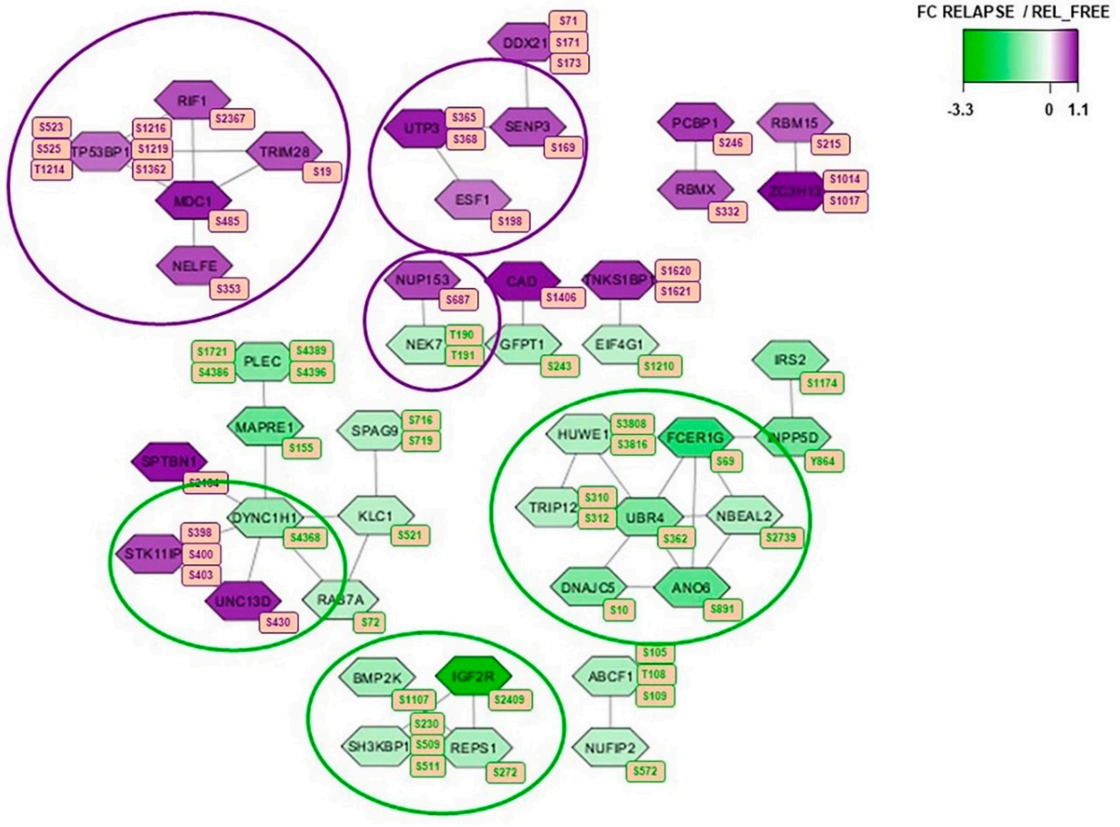

Figure S9. Phosphoproteins with differentially regulated phosphorylation sites in the RELAPSE vs. REL_FREE cohort dataset with unaltered overall expression. Networks of PPI based on STRING analysis of 107 phosphoproteins that were not significantly changed at the protein level, visualized in Cytoscape. The differentially regulated phosphorylation site(s) is shown next to each protein. FCs of phosphorylation are color-coded; purple-colored proteins showed a higher phosphorylation in the RELAPSE group and green-colored proteins showed a higher phosphorylation in the REL_FREE group. Encircled phosphoproteins networks represent phosphoproteins networks previously illustrated in Figure 3e in the main text.

(**a**)

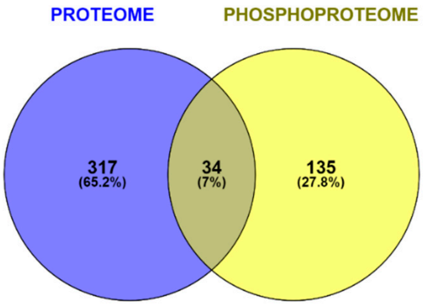

(**b**)

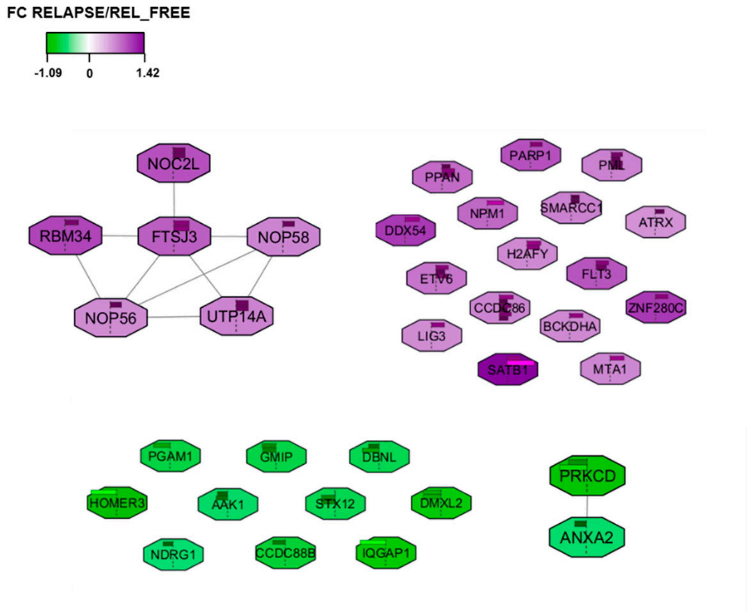

PHOSPHOSITES	
AAK1_S623	LIG3_S123
AAK1_T620	MTA1_S449
ANXA2_S26	NDRG1_S2
ATRX_S693	NOC2L_S672
BCKDHA_S337	NOC2L_S673
CCDC86_S113	NOP56_S519
CCDC86_S18	NOP58_S502
CCDC86_S58	NPM1_S125
CCDC86_S66	PARP1_S782
CCDC86_S91	PGAM1_S14
CCDC86_T65	PML_S36
CCDC88B_S1379	PML_S40
DBNL_S269	PML_S403
DBNL_T291	PML_S504
DDX54_S782	PPAN_S238
DMXL2_S944	PPAN_S240
DMXL2_S945	PPAN_S359
ETV6_S184	PRKCD_S304
ETV6_S22	PRKCD_S645
ETV6_T18	RBM34_S14
FLT3_S759	SATB1_S38
FLT3_S762	SATB1_S637
FTSJ3_S335	SMARCC1_S328
FTSJ3_S336	SMARCC1_S330
GMIP_S437	STX12_S139
GMIP_S441	STX12_S142
H2AFY_S170	UTP14A_S405
H2AFY_S173	UTP14A_S407
HOMER3_S159	ZNF280C_S80
IQGAP1_S1443	

Figure S10. Overlap of regulated proteins and phosphoproteins in the RELAPSE *vs* REL_FREE cohort study. (**a**) Venn diagram of significantly different proteins and phosphoproteins found in the proteome and phosphoproteome data analysis, respectively. (**b**) Cytoscape representation of the 34 proteins that are found differently abundant and phosphorylated in the proteome and phosphoproteome data analysis, respectively, i.e. at both protein and phosphosite level. Nodes are colored based on log_2 transformed FCs of protein expression levels; purple-colored proteins showed a higher expression in the RELAPSE group and green-colored proteins showed a higher expression in the REL_FREE group. Node heat strips show FC of the different phosphosites found in each protein and listed in the shown table.

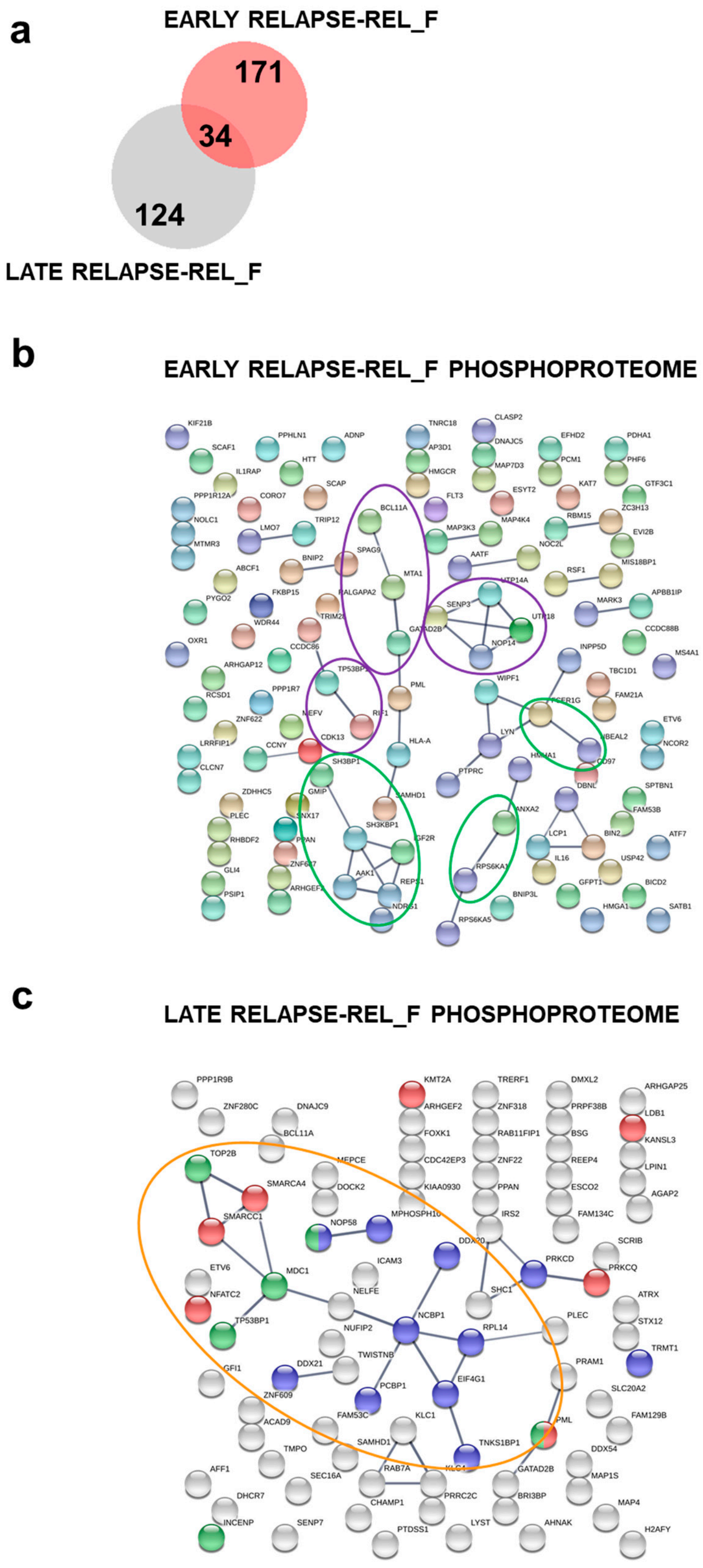

Figure S11. Comparison between the differentially regulated phosphorylation sites found in the EARLY RELAPSE vs. REL_FREE (EARLY RELAPSE-REL_F in the plots) and LATE RELAPSE vs. REL_FREE (LATE RELAPSE-REL_F in the plots) subcohorts. (**a**) Overlap of the differentially regulated phosphorylation sites found in the two subcohorts illustrated with a Venn diagram. (**b**) PPI network performed with STRING of the 171 phosphoproteins found in the EARLY RELAPSE vs.

REL_FREE subcohort. Components of phosphoprotein networks characterized in Figure 3e of the main text are highlighted here with a colored oval (purple for protein networks with higher phosphorylation in RELAPSE and green with higher phosphorylation in REL_FREE). Black edges mean confidence at 0.7 as interaction score. (**c**) PPI network performed with STRING of the 124 phosphoproteins found in the LATE RELAPSE vs. REL_FREE subcohort. A new network composed of RNA metabolism phosphoproteins (blue nodes), transcriptional regulators (red nodes) and SUMOylate target proteins (green nodes) is highlighted here with an orange oval.

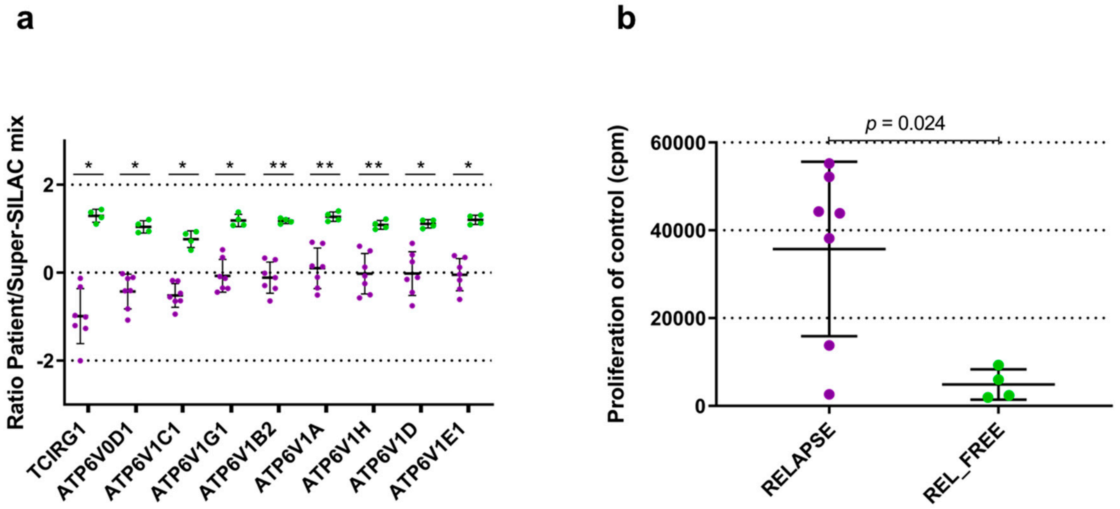

Figure S12. V-ATPase inhibitor assays. The proteomics results pointed to significantly different abundance of the proteins composing the V-ATPase complex. (**a**) For V-ATPase inhibitor testing, we selected four REL_FREE patients (P36, P44, P46 and P49) and seven RELAPSE patients (P9, P12, P15, P17, P18, P40 and P48) that had significantly higher and lower abundance of the V-ATPase subunits (according to the two-sample unequal variance *t*-test), respectively, in the super-SILAC proteomics experiment. The plot shows the relative protein expression (log_2 transformed ratio patient/super-SILAC mix) of the significantly different V-ATPase subunits quantified in each patient sample (* $p < 0.05$; ** $p < 0.01$). (**b**) The RELAPSE control cells had significantly higher proliferation than the REL_FREE cells.

a

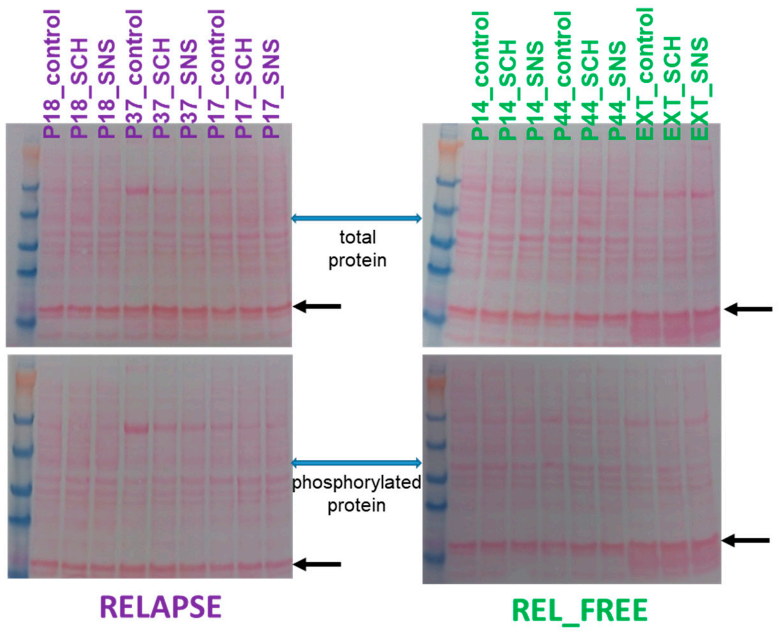

b

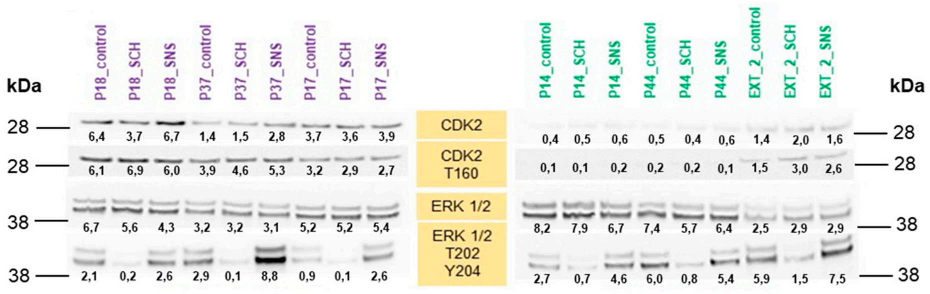

unpaired *t*-test	CDK2	CDK2 T160	ERK1/2	ERK1/2 T202 Y204
control	0,161	0,031	0,664	0,095
1000 nM SCH	0,095	0,067	0,625	0,077
400 nM SNS	0,083	0,048	0,478	0,649

Figure S13. Ponceau-stained membranes and Western blots. (**a**) Ponceau-stained membranes, used as loading controls (the arrows mark the 17kDa-band intensity taking for normalization purposes), and **b**) Western blots of sample lysates from primary cells of three RELAPSE and three REL_FREE patients untreated and treated with inhibitors against ERK1/2 (SCH: SCH772984) and CDK2/7/9 (SNS: SNS032). EXT refers to a REL_FREE patient outside the cohort used in this study. Western blots were not replicated. Band intensities of total and phosphorylated proteins were normalized before statistical analysis and intensity ratios from Table S6 are displayed under each band. RELAPSE and REL_FREE groups were compared using unpaired *t*-test. Grey cells show significance with $p < 0.05$.

Supplementary Tables

Table S1. Overview of the 41 AML patients used in this study.

Experimental patient code	BM blasts (%)	WBC count (10^9/L)	Gender	Age (years)	Clinical progression	Secondary AML	Time to relapse (months after diagnosis)	Survival time (months after diagnosis) (updated on 24/7/18)	Still alive (+)	Group	FAB classification	Cytogenetics
P2	90	19	M	29	CR1 - RELAPSE		12	12		RELAPSE	M4	46,XY
P3	25	6	M	56	CR1 - REL_FREE		-	120	death after 10 years (no relapse)	REL_FREE	M1	46,XY,inv(1)(p22?p34?),t(2;10?)(q33?;q22?),t(9;22)(q34;q11)
P4	53	45	M	60	CR1 - RELAPSE		4	4		RELAPSE		
P5	80	43	M	27	CR1 - Relapse - CR2 - SCT		11	274	+	RELAPSE	M2	46,XY
P6	95	10	F	66	CR1 - RELAPSE		16	19		RELAPSE	M1	46,XX
P9	84	27	M	46	CR1 - RELAPSE		27	39		RELAPSE	M1	46,XY
P10	89	11	M	57	CR1 - REL_FREE		-	96	died of another cancer	REL_FREE	M4	46,XY
P11	40	34	F	18	CR1 - Relapse - CR2 - SCT		11	89	+	RELAPSE	M4	46,XX,inv(16)(q13q22)
P12	66	25	F	46	CR1 - Relapse - CR2 - SCT		16	99	+	RELAPSE	M1	46,XX,inv(16)(p13q22)
P13	75	22	F	45	CR1 - REL_FREE	Chemotherapy	-	195	+	REL_FREE	M4	46,XX
P14	86	40	F	42	CR1 - REL_FREE		-	150	+	REL_FREE	M5	46,XX
P15	60	21	F	67	CR1 - RELAPSE		13	14		RELAPSE	M0	48,XX,+6,+21,inc[4]/46, XX[17]
P17	53	14	M	60	CR1 - RELAPSE		6	7		RELAPSE	M4	46,XY,del(9)(q13q33)[18]
P18	83	58	F	59	CR1 - RELAPSE	MDS	8	8		RELAPSE	M5	46,XX
P19	80	16	M	35	CR1 - Relapse - CR2 - SCT		44	135	+	RELAPSE	M2	46,XY
P20	93	46	F	55	CR1 - RELAPSE		12	12		RELAPSE	M0	46,XX
P21	99	114	F	48	CR1 - RELAPSE		6	11		RELAPSE	M0	
P22	30	4	M	41	CR1 - RELAPSE		26	42		RELAPSE	M4	46,XY
P23	97	170	M	46	CR1 - RELAPSE		11	11		RELAPSE	M4	46,XY
P24	50	31	M	53	CR1 - REL_FREE		-	250	+	REL_FREE	M4	46,XY
P26	83	59	M	53	CR1 - Relapse - CR2 - SCT		53	109	+	RELAPSE	M0	Multiple (+13 is dominating)
P29	83	71	M	58	CR1 - REL_FREE		-	114	+	REL_FREE	M5	46,XY
P30	92	10	M	67	CR1 - RELAPSE		5	49		RELAPSE	M0	45-46,XY,-5,+dic(1;5)(1p1?;5q13)
P31	86	38	F	36	CR1 - REL_FREE	Chemotherapy	-	232	+	REL_FREE	M5	46,XX,t(9;11)(p21;q23)
P32	95	47	M	62	CR1 - RELAPSE		6	16		RELAPSE	M1	
P34	76	14	M	48	CR1 - RELAPSE		6	7		RELAPSE	M5	46,XY
P36	32	33	M	36	CR1 - REL_FREE		-	70	+	REL_FREE	M4	46,XY,inv(16)(p13q22)
P37	47	65	F	57	CR1 - RELAPSE		12	12		RELAPSE	M4	46,XX,inv(16)(13;q22)[5]/46,idem,t(X;6)(p22;p12)[13]/46,XX[2]
P40	95	48	F	63	CR1 - RELAPSE		4	6		RELAPSE	M1	46,XX,dup(11)(q23.3q23.3)
P42	90	351	M	43	CR1 - REL_FREE		-	253	+	REL_FREE	M5	46,XY,inv(16)
P43	52	50	M	48	CR1 - REL_FREE		-	170	+	REL_FREE	M4	46,XY,inv(16)(p13q22)
P44	77	101	M	65	CR1 - REL_FREE		-	109	+	REL_FREE	M5	Multiple
P45	95	24	F	61	CR1 - Relapse - CR2 - SCT	MDS	12	97	death by toxicity/infection	RELAPSE	M5	46,XX
P46	83	37	F	60	CR1 - REL_FREE		-	109	+	REL_FREE	M5	46,XX
P47	55	9	F	41	CR1 - REL_FREE		-	159	+	REL_FREE	M4	46,XX
P48	72	28	M	68	CR1 - RELAPSE		3	23		RELAPSE	M1	46,XY
P49	80	29	M	54	CR1 - REL_FREE	Chemotherapy	-	174	+	REL_FREE	M5	46,XY
P50	95	182	F	36	CR1 - RELAPSE		4	12		RELAPSE	M4	46,XX
P51	95	112	F	32	CR1 - Relapse - CR2 - SCT		12	142	+	RELAPSE	M5	46,XX,del(5)(q31q34)[5]/46,XX[15]
P52	25	33	M	62	CR1 - RELAPSE		8	9		RELAPSE	M4	47,XY,+8[12]/46,XY[13]
P53	50	65	M	48	CR1 - REL_FREE		-	286	+	REL_FREE	M4	46,XY

Gene columns of the table (by group):

NPM1	SIGNALING						TUMOR SUPRESSORS				DNA METHYLATION					MYELOID TF			CHROMATIN MODIFICATION			COHESIN		SPLICEOSOME/TR			OTHERS	
NPM1 Ins	FLT3-ITD	FLT3-TKD	NRAS	HRAS	KIT	PTPN11	IKZF1	CDKN2A	PHF6	WT1	DNMT3A	TET2	IDH1	IDH2	KMT2A	RUNX1	CEPBA	GATA2	ASXL1	EZH2	KDM6A	RAD21	STAG2	BCORL1	BCOR	SRSF2	CALR	CSF3R

Gene cell colors indicate: light yellow = wild type; red = mutated; grey = not determined; Other grey colored cells on the FAB classification and Cytogenetics columns indicate uncertain or not determined data; TF/TR: transcription factors/repressors; BM: bone marrow; WBC: white blood cells; CR: complete remission; NPM1 Ins: a 4 bp-insertion/duplication; SCT: stem-cell transplantation; ITD: internal tandem duplication; FAB: French-American-British; TKD: tyrosine kinase domain.

Table S2. Overview of the nine AML patients external to the study cohort used for cell proliferation and Western blot assays.

Experimental patient code	BM blasts (%)	WBC count (10^9/L)	Gender	Age (years)	Clinical progression	Secondary AML	Time to relapse (months after diagnosis)	Survival time (months after diagnosis) (updated on 31/05/19)	Still alive (+)	Group	FAB classification	NPM1 Ins	FLT3-ITD	CEBPA	Cytogenetics
REL_F_EXT_1	48	28.2	F	33	CR1 - REL_FREE		-	50	+	REL_FREE	M1				t(8;21)
REL_F_EXT_2	94	72.4	M	32	CR1 - REL_FREE		-	35	+	REL_FREE	M2				46, XY
REL_F_EXT_3	77	241	M	42	CR1 - AlloSCT in CR1		-	81	+	REL_FREE	M2				46, XY
REL_F_EXT_4	61	131	M	19	CR1 - AlloSCT in CR1		-	58	+	REL_FREE	M5				46, XY
REL_F_EXT_5	28	34.5	M	48	CR1 - AlloSCT in CR1		-	48	+	REL_FREE	M4				46, XY
REL_EXT_1	59	34.2	M	61	CR1 - RELAPSE		10	17		RELAPSE	M4				46, XY
REL_EXT_2	90	59.8	M	42	CR1 - RELAPSE		5	7		RELAPSE	M5				46, XY
REL_EXT_3	95	58.3	M	64	CR1 - RELAPSE		7	8		RELAPSE	M2				46, XY
REL_EXT_4	70	96.9	F	46	CR1 - RELAPSE		27	29		RELAPSE	M2				iso13

Cell coloring and abbreviations are the same as in Table S1.

Table S3. List of 14 transcription-related proteins and/or phosphoproteins with higher abundance/phosphorylation in the RELAPSE group subjected to ChIP analysis to determine binding sites of target genes.

Transcription protein	Biological function	ENCODE accession ID
AFF1	DNA-binding transcription	ENCFF452EKB, ENCFF267LVE
ATRX	Transcription regulation Chromatin remodeling	N/A
BBX	DNA-binding transcription	N/A
BCL11A	DNA-binding transcription	N/A
CEBPZ	DNA-binding transcription	ENCFF002EEI
ETV6	DNA-binding transcription	ENCFF696VXH
GATAD2B	Transcription regulation	N/A
IKZF1	DNA-binding transcription	ENCFF015LUT, ENCFF424OOE
IRF5	DNA-binding transcription	N/A
PARP1	Chromatin remodeling	N/A
PML	DNA-binding transcription	ENCFF000QDH
SATB1	Transcription regulation Chromatin remodeling	N/A
SMARCC1	Chromatin remodeling	N/A
TRIM28	Transcription regulation Chromatin remodeling	ENCFF000QJK

ChIP data was retrieved from ENCODE and from experiments using K562, a human undifferentiated myeloid cell line. For some proteins, ChIP-seq data were not found or did not successfully pass the audit status of the computation pipeline (indicated as N/A).

Table S4. List of overlapping regulated proteins and phosphoproteins in the 26 RELAPSE and 15 REL_FREE patient cohort.

	Proteome	PHOSPHO-1	PHOSPHO-2	PHOSPHO-3	PHOSPHO-4	PHOSPHO-5	PHOSPHO-6
Gene	FC REL/REL_F	FC REL/REL_F	FC REL/REL_F	FC REL/REL_F	FC REL/REL_F	FC REL/REL_F	FC REL/REL_F
SATB1	1.42	1.00	1.93				
ZNF280C	1.10	0.90					
DDX54	1.10	1.04					
RBM34	1.04	0.81					
PARP1	0.97	0.87					
NOC2L	0.97	0.72	0.72				
FLT3	0.95	0.91	0.75				
FTSJ3	0.90	0.92	0.92				
NPM1	0.85	1.21					
PPAN	0.83	0.60	0.93	0.93			
ETV6	0.78	0.89	0.65	0.90			
SMARCC1	0.71	0.59	0.59				
PML	0.71	0.81	0.65	0.81	0.78		
UTP14A	0.70	0.74	0.74				
NOP58	0.69	0.69					
CCDC86	0.69	1.01	0.59	0.86	0.59	0.86	0.63
MTA1	0.68	1.03					
H2AFY	0.66	1.01	0.92				
BCKDHA	0.66	0.97					
NOP56	0.65	0.67					
LIG3	0.65	0.96					
ATRX	0.61	0.66					
NDRG1	-0.58	-0.72					
ANXA2	-0.60	-0.67					
AAK1	-0.62	-0.74	-0.82				
STX12	-0.69	-0.81	-0.98	-0.98			
DBNL	-0.69	-0.75	-1.21				
GMIP	-0.73	-1.00	-0.95				
PGAM1	-0.74	-1.20					
CCDC88B	-0.78	-0.89					
DMXL2	-0.88	-1.27	-1.27				
IQGAP1	-0.92	-1.84					
HOMER3	-1.10	-1.86					
PRKCD	-1.03	-1.06	-1.58				

RELAPSE/REL_FREE FCs of the proteins (Proteome) and of different phosphosites (PHOSPHO-1 TO PHOSPHO-6) in the phosphoproteins are shown. Positive and negative FCs are shown in purple and green, respectively.

Table S5. Cell proliferation assays using kinase inhibitors.

Inhibitor(s)	C1 (nM)	C2 (nM)	*P* value RELAPSE	*P* value REL_FREE
CX	5000		.169	.051
CX	15000		.005	.008
AB	250		.005	.008
AB	500		.005	.008
SNS	200		.013	.008
SNS	400		.005	.008
SCH	500		.005	.008
SCH	1000		.005	.008
CX+AB	1500	50	.203	.008
CX+SNS	1500	50	.017	.008
AB+SNS	50	50	.059	.008
AB+SCH	50	125	.007	.008
SNS+SCH	50	125	.037	.086

List of inhibitors tested in proliferation assays with primary cells from RELAPSE (P11, P17, P18, P22, P37 and P45 from the original cohort and four patient samples from an external cohort) and REL_FREE patients (P14, P36, P46 and P47 from the original cohort and five patient samples from an external cohort). *P* values are calculated after Wilcoxon matched pairs (control and treatment) test for each patient group. Underlined concentrations (C) were used for the preparation of samples for Western blot analysis (Figure S13). CX: CX-4945, an inhibitor against CSK2; AB: Abemaciclib, an inhibitor against CDK4/6; SNS: SNS032, an inhibitor against CDK2/7/9; SCH: SCH772984, an inhibitor against ERK1/2.

Table S6. Densitometry intensities and normalized intensities of Western blot bands.

peak CDK2	area	area _control band	area/area_control band	peak CDK2 T160	area	area _control band	area/area_control band
P18_control	6983,033	1092,527	6,392	P18_control	11982,012	1956,820	6,123
P18_SCH	5074,941	1389,770	3,652	P18_SCH	12095,941	1758,648	6,878
P18_SNS	10647,891	1590,648	6,694	P18_SNS	9367,305	1564,698	5,987
P37_control	2126,891	1471,234	1,446	P37_control	7206,648	1838,234	3,920
P37_SCH	2704,941	1795,062	1,507	P37_SCH	8175,184	1771,477	4,615
P37_SNS	4859,134	1718,355	2,828	P37_SNS	8415,598	1576,770	5,337
P17_control	4424,648	1196,234	3,699	P17_control	4615,497	1444,891	3,194
P17_SCH	4848,770	1338,648	3,622	P17_SCH	4657,184	1584,527	2,939
P17_SNS	5503,669	1419,770	3,876	P17_SNS	4653,669	1723,820	2,700
P14_control	644,234	1476,698	0,436	P14_control	51,364	1158,799	0,044
P14_SCH	736,527	1537,548	0,479	P14_SCH	54,657	883,577	0,062
P14_SNS	850,820	1408,355	0,604	P14_SNS	159,021	859,870	0,185
P44_control	547,870	1045,234	0,524	P44_control	177,899	773,527	0,230
P44_SCH	665,406	1482,648	0,449	P44_SCH	214,263	994,284	0,215
P44_SNS	746,284	1199,941	0,622	P44_SNS	106,192	819,527	0,130
EXT_2_control	2123,941	1492,406	1,423	EXT_2_control	1454,113	999,477	1,455
EXT_2_SCH	2967,941	1498,406	1,981	EXT_2_SCH	3068,477	1038,648	2,954
EXT_2_SNS	2663,477	1712,062	1,556	EXT_2_SNS	3018,426	1167,234	2,586

peak erk1/2	area	area _control band	area/area_control band	peak erk1/2 T202 Y204	area	area _control band	area/area_control band
P18_control	7328,770	1092,527	6,708	P18_control	4183,042	1956,820	2,138
P18_SCH	7743,477	1389,770	5,572	P18_SCH	292,263	1758,648	0,166
P18_SNS	6791,648	1590,648	4,270	P18_SNS	4054,648	1564,698	2,591
P37_control	4761,113	1471,234	3,236	P37_control	5297,527	1838,234	2,882
P37_SCH	5754,477	1795,062	3,206	P37_SCH	219,263	1771,477	0,124
P37_SNS	5361,527	1718,355	3,120	P37_SNS	13905,598	1576,770	8,819
P17_control	6244,355	1196,234	5,220	P17_control	1265,376	1444,891	0,876
P17_SCH	6941,113	1338,648	5,185	P17_SCH	70,607	1584,527	0,045
P17_SNS	7618,841	1419,770	5,366	P17_SNS	4495,477	1723,820	2,608
P14_control	12070,012	1476,698	8,174	P14_control	3169,497	1158,799	2,735
P14_SCH	12150,234	1537,548	7,902	P14_SCH	598,205	883,577	0,677
P14_SNS	9395,820	1408,355	6,671	P14_SNS	3913,527	859,870	4,551
P44_control	7708,820	1045,234	7,375	P44_control	4662,820	773,527	6,028
P44_SCH	8500,062	1482,648	5,733	P44_SCH	765,083	994,284	0,769
P44_SNS	7734,820	1199,941	6,446	P44_SNS	4451,527	819,527	5,432
EXT_2_control	3771,355	1492,406	2,527	EXT_2_control	5932,527	999,477	5,936
EXT_2_SCH	4418,820	1498,406	2,949	EXT_2_SCH	1563,698	1038,648	1,506
EXT_2_SNS	5046,820	1712,062	2,948	EXT_2_SNS	8727,355	1167,234	7,477

Data intensities obtained using ImageJ correspond to the 3 RELAPSE and 3 REL_FREE patient samples shown in Figure S13. Normalized data were used for statistical analysis between the groups.

Supplementary References

1. Mayer, R.J.; Davis, R.B.; Schiffer, C.A.; Berg, D.T.; Powell, B.L.; Schulman, P.; Omura, G.A.; Moore, J.O.; McIntyre, O.R.; Frei, E., 3rd. Intensive postremission chemotherapy in adults with acute myeloid leukemia. Cancer and Leukemia Group B. *N. Engl. J. Med.* **1994**, *331*, 896–903, doi:10.1056/NEJM199410063311402.
2. Kelstrup, C.D.; Jersie-Christensen, R.R.; Batth, T.S.; Arrey, T.N.; Kuehn, A.; Kellmann, M.; Olsen, J.V. Rapid and deep proteomes by faster sequencing on a benchtop quadrupole ultra-high-field Orbitrap mass spectrometer. *J. Proteome Re.s* **2014**, *13*, 6187–6195, doi:10.1021/pr500985w.
3. Cox, J.; Hein, M.Y.; Luber, C.A.; Paron, I.; Nagaraj, N.; Mann, M. Accurate proteome-wide label-free quantification by delayed normalization and maximal peptide ratio extraction, termed MaxLFQ. *Mol. Cell Proteomics* **2014**, *13*, 2513–2526, doi:10.1074/mcp.M113.031591.
4. Bantscheff, M.; Schirle, M.; Sweetman, G.; Rick, J.; Kuster, B. Quantitative mass spectrometry in proteomics: a critical review. *Anal. Bioanal. Chem.* **2007**, *389*, 1017–1031, doi:10.1007/s00216-007-1486-6.

© 2020 by the authors. Licensee MDPI, Basel, Switzerland. This article is an open access article distributed under the terms and conditions of the Creative Commons Attribution (CC BY) license (http://creativecommons.org/licenses/by/4.0/).
